# Effects of Brain Atlases and Machine Learning Methods on the Discrimination of Schizophrenia Patients: A Multimodal MRI Study

**DOI:** 10.3389/fnins.2021.697168

**Published:** 2021-07-27

**Authors:** Jinyu Zang, Yuanyuan Huang, Lingyin Kong, Bingye Lei, Pengfei Ke, Hehua Li, Jing Zhou, Dongsheng Xiong, Guixiang Li, Jun Chen, Xiaobo Li, Zhiming Xiang, Yuping Ning, Fengchun Wu, Kai Wu

**Affiliations:** ^1^Department of Biomedical Engineering, School of Material Science and Engineering, South China University of Technology, Guangzhou, China; ^2^Guangdong Engineering Technology Research Center for Translational Medicine of Mental Disorders, Guangzhou, China; ^3^National Engineering Research Center for Tissue Restoration and Reconstruction, South China University of Technology, Guangzhou, China; ^4^The Affiliated Brain Hospital of Guangzhou Medical University, Guangzhou Huiai Hospital, Guangzhou, China; ^5^Guangdong Engineering Technology Research Center for Diagnosis and Rehabilitation of Dementia, Guangzhou, China; ^6^National Engineering Research Center for Healthcare Devices, Guangzhou, China; ^7^Department of Biomedical Engineering, New Jersey Institute of Technology, Newark, NJ, United States; ^8^Department of Radiology, Panyu Central Hospital of Guangzhou, Guangzhou, China; ^9^Key Laboratory of Biomedical Engineering of Guangdong Province, South China University of Technology, Guangzhou, China; ^10^Department of Nuclear Medicine and Radiology, Institute of Development, Aging and Cancer, Tohoku University, Sendai, Japan

**Keywords:** multimodal MRI, schizophrenia, brain atlas, machine learning, classification

## Abstract

Recently, machine learning techniques have been widely applied in discriminative studies of schizophrenia (SZ) patients with multimodal magnetic resonance imaging (MRI); however, the effects of brain atlases and machine learning methods remain largely unknown. In this study, we collected MRI data for 61 first-episode SZ patients (FESZ), 79 chronic SZ patients (CSZ) and 205 normal controls (NC) and calculated 4 MRI measurements, including regional gray matter volume (GMV), regional homogeneity (ReHo), amplitude of low-frequency fluctuation and degree centrality. We systematically analyzed the performance of two classifications (SZ vs NC; FESZ vs CSZ) based on the combinations of three brain atlases, five classifiers, two cross validation methods and 3 dimensionality reduction algorithms. Our results showed that the groupwise whole-brain atlas with 268 ROIs outperformed the other two brain atlases. In addition, the leave-one-out cross validation was the best cross validation method to select the best hyperparameter set, but the classification performances by different classifiers and dimensionality reduction algorithms were quite similar. Importantly, the contributions of input features to both classifications were higher with the GMV and ReHo features of brain regions in the prefrontal and temporal gyri. Furthermore, an ensemble learning method was performed to establish an integrated model, in which classification performance was improved. Taken together, these findings indicated the effects of these factors in constructing effective classifiers for psychiatric diseases and showed that the integrated model has the potential to improve the clinical diagnosis and treatment evaluation of SZ.

## Introduction

Schizophrenia (SZ) is a chronic psychiatric disorder, characterized by disabling mental symptoms such as auditory delusions, hallucinations and disrupted higher-order cognitive functions (Austin, 2005; Leucht et al., 2007). With the development of machine learning methods, both structural and functional magnetic resonance imaging (MRI) data have been applied into the discriminative analyses of SZ patients (Kasparek et al., 2011; Deanna et al., 2012; Ota et al., 2012; Liu Y. et al., 2017; Chen et al., 2020). For example, support vector machine (SVM) is the most widely used method to distinguish SZ patients from normal controls (NCs) (Liu Y. et al., 2017; Chen et al., 2020) or to differentiate illness stages of SZ, such as first-episode schizophrenia (FESZ) and chronic schizophrenia (CSZ) (Lu et al., 2018; Wu et al., 2018). Similarly, other classifiers such as random forest (Deanna et al., 2012) and linear discriminant analysis (LDA) (Kasparek et al., 2011; Ota et al., 2012) have also been utilized in discriminative analyses of SZ patients.

Recently, a number of discriminative studies of SZ patients have adopted the strategy of multiple classifiers, including LDA (Junhua et al., 2018), SVM (Watanabe et al., 2014; Raymond et al., 2017) and extreme learning machine (Iqbal et al., 2017), and multiple dimensionality reduction algorithms, such as principle component analysis (PCA) (Raymond et al., 2017) and *t*-test (Junhua et al., 2018). Importantly, different classifiers have been selected as the best classifier in previous studies, which shows diversity among the machine learning methods. Thus, to achieve an optimal performance of a discriminative analysis, a systematic evaluation with multiple machine learning methods is essential and of great importance.

Moreover, previous discriminative analyses using different brain atlases have shown that the choice of brain atlases seems rather arbitrary and could lead to different results (Kalmady et al., 2019). A number of researchers have performed discriminative analyses of SZ patients based on the automated anatomical labeling (AAL) atlas (Tzourio-Mazoyer et al., 2002) with accuracies from 76.3% to 85% (Longfei et al., 2013; Kim et al., 2015; Junhua et al., 2018; Matsubara et al., 2019). A brain atlas with 95 regions of interest (ROIs) has also been utilized in the discriminative analysis of SZ patients to achieve 89.3% sensitivity and 93.6% specificity (Karageorgiou et al., 2011). Additionally, another study used the Desikan atlas for discriminative analysis and obtained an accuracy of 85.0% (Xiao et al., 2017). However, few studies have evaluated the effects of brain atlases on discriminative analyses of SZ patients.

In this study, we collected structural MRI (sMRI) and resting-state functional MRI (rs-fMRI) data from 345 subjects and used three brain atlases to calculate 4 MRI measurements, including regional gray matter volume (GMV), regional homogeneity (ReHo), amplitude of low-frequency fluctuation (ALFF) and degree centrality (DC). We then performed a systematic evaluation of the classification performances in two classifications (NC vs SZ, FESZ vs CSZ) using five classifiers, two cross validation methods, and 3 dimensionality reduction algorithms. Moreover, an ensemble learning method was performed to establish an integrated model to improve the clinical diagnosis.

## Materials and Methods

### Subjects

A total of 61 FESZ patients, 79 CSZ patients and 205 NCs were included (Table 1). The inclusion and exclusion criteria of subjects were the same as those in our previous studies (Lu et al., 2016; Wu et al., 2018). All subjects, aged 18 to 45 and of Han nationality, underwent a clinical assessment with the Positive and Negative Syndrome Scale (PANSS) which contains three subscales (general psychopathology, positive symptoms and negative symptoms) and indicates the severity of the symptoms (Kay et al., 1987; Van Tol et al., 2014). Only the subjects with PANSS scores over 60 and with a period of education of more than 6 years were chosen for the project. Meanwhile, they also had to be diagnosed by experienced clinical psychiatrists to be SZ in accordance with the Diagnostic and Statistical Manual of Mental Disorders-IV-Text Revision (DSM-IV-TR) criteria (First et al., 1997). Among these subjects, first-episode patients with a course of disease under 2 years were categorized as FESZ if they had not taken any antipsychotic drugs. Meanwhile, the patients who had suffered recurrent symptoms and had already undergone drug therapy with a course of disease over 2 years were categorized as CSZ.

**TABLE 1:** Demographic and clinical characteristics.

	**FESZ patients**	**CSZ patients**	**NC**	**Statistic value**	***P*-value**
Gender (M/F)	41/20	54/25	110/95	χ^2^=3.53	0.03
Age (years)	32.08 ± 7.42	33.21 ± 8.37	32.52 ± 8.40	*F* = 5.39	<0.05^a,b^
Education (years)	10.39 ± 3.25	11.97 ± 3.22	12.84 ± 2.83	*F* = 21.33	<0.05^a,b^
PANSS-PScore	24.02 ± 4.50	22.47 ± 5.70	–	*T* = 1.74	0.083
PANSS-NScore	21.64 ± 7.70	23.22 ± 7.29	–	*T* = −1.24	0.218
PANSS-GScore	40.31 ± 8.85	39.54 ± 10.18	–	*T* = 0.47	0.641
PANSS-TScore	85.97 ± 17.49	85.23 ± 19.44	–	*T* = 0.23	0.816

*All data presented above were in format: average ± standard deviation. The factor age (years) and education (years) were analyzed by separate one-way ANOVA and the gender was analyzed by χ^2^ test. *Post hoc* pairwise comparison was utilized to analyze distinguished discrimination with two-sample *t*-test. *P-*value < 0.05 was considered significant. ^*a*^*Post hoc* pairwise comparison showed the significant discrepancy between FESZ and NC. ^*b*^*Post hoc* pairwise comparison showed the significant discrepancy between CSZ and NC.*

*CSZ, chronic schizophrenia; F, female; FESZ, first-episode schizophrenia; GScore, general score; M, male; NCs, normal controls; NScore, negative syndrome score; PScore, positive syndrome score; TScore, total syndrome score.*

If one of the following criteria were met, the SZ patient was excluded: (1) alcohol dependence or other mental disorders, such as depressive disorder, dementia or ental retardation, based on DSM-IV-TR criteria; (2) severe physical disorders potentially derived from substance dependence including definite diabetes, hypertension, heart disease, thyroid diseases or narrow-angle glaucoma; (3) history of epilepsy or febrile convulsions; (4) electroconvulsive therapy within the past six months; (5) serious tardive dyskinesia or drug-induced neuroleptic malignant syndrome; (6) contraindication for MRI; (7) lack of legal guardians or noncompliant with drug administration; (8) an irritative state or a serious suicide attempt; and (9) lactation, pregnancy or anticipated pregnancy. Meanwhile, NCs with pregnancy, contraindications for MRI or relatives diagnosed with psychiatric Axis I disorders based on the DSM-IV-ST criteria were also excluded. All the subjects’ data were collected from the Affiliated Brain Hospital of Guangzhou Medical University, and all subjects were informed about the experimental details and signed informed consent before clinical tests. The research was strictly subject to the Declaration of Helsinki and was under approval of the ethics committees of the Affiliated Brain Hospital of Guangzhou Medical University.

### MRI Data Acquisition

Magnetic resonance imaging data of all subjects were collected by a Philips 3T MR device system in the Affiliated Brain Hospital of Guangzhou Medical University. The echo-planar imaging (EPI) sequence (repetition time = 2,000 ms, echo time = 30 ms, acquisition time = 2,000 ms, field of view = 210 mm × 210 mm, flip angle = 90°, spatial resolution = 3.4 mm × 3.4 mm × 4 mm, 64 × 64 × 33 matrix) was used to generate the functional MRI data. The gradient-echo T1-weighted sequence (repetition time = 8.2 ms, echo time = 3.7 ms, flip angle = 7°, spatial resolution = 1 mm × 1 mm × 1 mm, 256 × 256 × 188 matrix) was used to generate the structural MRI data. All participants were instructed to minimize head movement with the eyes closed in a sober state.

### Preprocessing

The sMRI data were preprocessed by the SPM12 software package^1^ to calculate GMV. The raw images were first standardized with a customized template provided by the DARTEL template creation tool to eliminate the deviation caused by individual discrepancies. Then they were separated into gray matter (GM), white matter (WM) and cerebrospinal fluid (CSF) by the VBM toolkit embedded in the SPM12 software package. The images were also smoothed by an 8 mm full width at half maximum (FWHM) Gaussian kernel. Finally, the structural brain data from GM images were calculated in each region of three brain atlases, including the AAL atlas with 90 brain regions (Tzourio-Mazoyer et al., 2002), the human brainnetome (HBN) atlas with 246 brain regions (Fan et al., 2016), and the groupwise whole-brain (GWB) atlas with 268 brain regions (Shen et al., 2013; Finn et al., 2015).

The rs-fMRI data were also preprocessed by SPM12 and DPARSF V4.4 software^2^. The image data from the first 10 time series were excluded owing to the instability of the device and fluctuations in the subjects’ mental state at the beginning. The noise from the variance in signal acquisition times and from head motion was eliminated to amplify the valid image signal. The images were then normalized by the standard EPI template. Finally, the bandpass filter (0.01–0.08 Hz) was utilized to reduce the high-frequency physiological noise and the low-frequency drift. Three rs-fMRI measurements including ReHo, ALFF and DC were calculated in each region of the three brain atlases.

ReHo is Kendall’s coefficient of concordance on the time series of a certain voxel with respect to its 26 adjacent neighbors, suggesting functional synchronization within the voxel and its neighbors (Govindarajulu, 1992). The ReHo values were normalized to lessen the deviation resulting from individual variance and were averaged in each region of the different brain atlases. ALFF is measured as the averaged square root within the bandpass (0.01–0.08 Hz) after fast Fourier transform (FFT) for each voxel and represents the level of regional spontaneous neuronal activity (Yu-Feng et al., 2007). Similarly, the ALFF values of each voxel were divided by the global average ALFF value for normalization and averaged in each region of the different brain atlases. The DC is described as the average of the Pearson correlation coefficients between the time series of a certain ROI and those of other ROIs, evaluating the connection degree of a certain ROI to other ROIs (Zang et al., 2004). The time series of a certain ROI was calculated as the averaged time series of all voxels in that region.

### Classification Analysis

After the preprocessing, the multimodal features were combined to form the concatenated feature vector. The concatenated feature vector was composed of 360 measurements (90 GMV measurements+90 ReHo measurements+90 ALFF measurements+90 DC measurements) if AAL atlas was used, of 986 measurements (246 GMV measurements+246 ReHo measurements+246 ALFF measurements+246 DC measurements) if HBN atlas was used, and of 1,072 measurements (268 GMV measurements+268 ReHo measurements+268 ALFF measurements+268 DC measurements) if GWB atlas was used.

**FIGURE 1:**
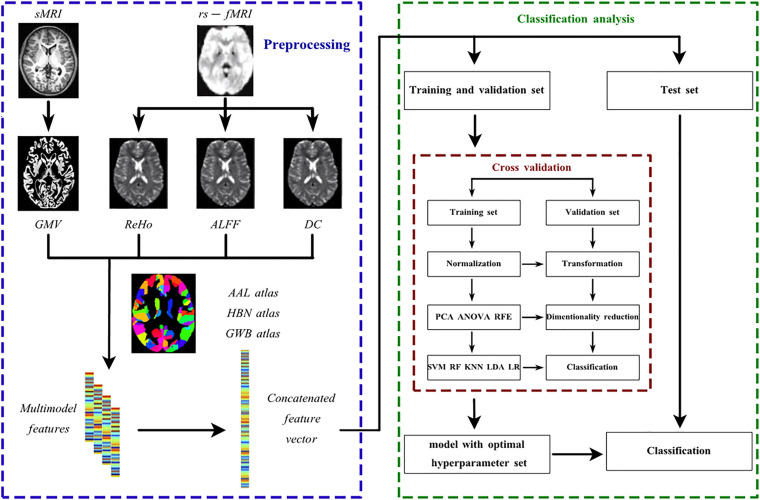
The pipeline architecture of the data preprocessing and discriminative analysis. AAL atlas, automated anatomical labeling atlas; ALFF, amplitude of low frequency fluctuation; ANOVA, analysis of variance; DC, degree centrality; GMV, regional gray matter volume; GWB atlas, groupwise whole-brain atlas; HBN atlas human brainnetome atlas; KNN, K nearest neighbor; LDA, linear discriminant analysis; LR, logistic regression; PCA, principle component analysis; ReHo, regional homogeneity; RF, random forest; RFE, recursive feature elimination; rs-fMRI, resting-state functional magnetic resonance imaging; sMRI, structural magnetic resonance imaging; SVM, support vector machine.

With the concatenated feature vector available, the whole pipeline architecture of classification is shown in Figure 1 In both classifications, five classifiers were utilized, including SVM, logistic regression (LR), LDA, random forest (RF) and K nearest neighbor (KNN). First developed by Vapnick in 1995, SVM aims to find the optimal hyperplane separating the dataset with different labels into multiple hyperspaces (Sain, 1997). Similar to SVM, LR also generates a hyperplane by a linear transformation function and sigmoid activation function to separate the data and to further provide the probability of unseen data being classified into a certain group (Peng and Ingersoll, 2002). LDA was first suggested by Fisher in 1936 (Fisher, 1936). Its principle is to project the dataset to the 1D dimension where the points representing data within the same group tend to get close and points representing data in different groups separate from each other (Fisher, 1936). RF is a bagging algorithm, and it predicts unseen data labels based on votes from all decision trees embedded in RF (Breiman, 1996). KNN simply counts the labels of a datum’s K nearest neighbors and predicts this datum’s label as the one with the highest frequency (Zhang, 2016). The list of hyperparameters available for all classifiers is shown in Supplementary Table 1. The best hyperparameter set was selected by cross validation as mentioned later.

Considering the redundancy or irrelevance of the features which may lead to overfitting when training, dimensionality reduction was performed before classification process. The dimensionality reduction algorithms applied in this study included PCA, analysis of variance (ANOVA) and recursive feature elimination (RFE). PCA has been widely used as a feature selection method in machine learning classification (Cao et al., 2003; Kriti Virmani et al., 2016). It basically projects the data to lower dimensions with the largest variance by a linear function (Jolliffe, 2005). it calculates the eigenvalues and eigenvectors of the covariance matrix in original feature space, and then selects n% percent of eigenvalues to represent the discriminative energy of data on different level. The corresponding n% percent of eigenvectors would then form the transformation matrix. ANOVA is also a common method for feature selection (Sheikhan et al., 2013; Bejani and Gharavian, 2014; Li et al., 2018; Abdulsalam et al., 2020). It first performs the F test on each separate feature together with data labels. Then it selects features according to the percentile of the highest F scores (Neter et al., 1996). Similarly, RFE also excludes the features with low relevance to label prediction, but the criteria refer to the weights derived from a certain classifier such as SVM (Guyon et al., 2002). Basically, it prunes the least important features iteratively according to the weights derived from the classifier until the desired number of features to select is reached. It has also been widely used in feature selection and achieved good results (Fernandez-Lozano et al., 2014; Xue et al., 2018; Albashish et al., 2021). The list of hyperparameters available for all dimensionality reduction methods is shown in Supplementary Table 2. The best hyperparameter set was selected by cross validation as mentioned below.

Twenty percent of the whole dataset was randomly separated to establish a separate test set for classification evaluation after training, and the rest of the dataset was subjected to cross validation to construct the predictive model with the best hyperparameter set. The application of a separate test set guaranteed the generalization of the classification model. In this study, there were two types of cross validation methods available, namely, 10-fold cross validation (10FCV) and leave-one-out cross validation (LOOCV). The cross validation in the study is mainly used for hyperparameter selection, which is also known as grid search cross validation (Sarah and Media, 2017). In 10FCV, the dataset was split into 10 portions of equal size, where 1 portion was used for validation and the remaining nine portions were used for training, and this occurred in an iterative manner. During the cross validation, the data were standardized by removing the mean and scaling to unit variance before the dimensionality reduction and classification. The normalization, dimensionality reduction and classification constituted the model as a whole. All possible combinations of hyperparameters for the model, as shown in Supplementary Tables 1, 2, were validated by the 10 validation sets in the 10FCV. The optimal hyperparameter set for the model was selected based on the average accuracy generated from the 10 iterations and was applied to construct the predictive model trained by 10 portions of data taken together. The performance of the model was assessed using the separate test set. Similar to 10FCV, LOOCV simply selects one portion of the data for validation and all others for training.

The classification performances were systematically analyzed in both classifications for different combinations of brain atlases, classifiers, cross validation methods, and dimensionality reduction algorithms. The receiver operating characteristic (ROC) curve was also plotted to calculate the area under the ROC curve (AUC), which was between 0 and 1. It is believed that the closer the AUC is to 1, the better the classification is. In parallel, the sensitivity and specificity were measured for a further assessment of the performance, and these definitions are shown below.

(1) $$S\,e\,n\,s\,i\,t\,i\,v\,i\,t\,y=\frac{T\,P}{T\,P+F\,N}$$

(2) $$S\,p\,e\,c\,i\,f\,i\,c\,i\,t\,y=\frac{T\,N}{T\,N+F\,P}$$

(3) $$A\,c\,c\,u\,r\,a\,c\,y=\frac{T\,P+T\,N}{T\,N+F\,N+T\,N+T\,P}$$

True positive (TP): the number of positive samples predicted as positive; true negative (TN): the number of negative samples predicted as negative; false positive (FP): the number of negative samples predicted as positive; and false negative (FN): the number of positive samples predicted as negative.

Furthermore, the permutation test, a widely used nonparametric test examining a null hypothesis (Golland and Fischl, 2003; Liu et al., 2015), was performed to analyze the significance of the classification results. In this study, the permutation test was carried out by randomly permutating the labels of all datasets and evaluating the classification performance with these permutated data 1,000 times. If the *P*-value was small enough (*P* < 0.05 was used in this study), the hypothesis that the classifier had significantly discovered the difference between the two groups with given set of imaging data could be safely accepted. The *P*-value was calculated as the percentage of classifications with better performance based on permutated data over all 1,000 trials.

Subsequently, the parameters from the best model (i.e., the combination of a certain dimensionality reduction algorithm and classifier) were analyzed to discover the brain regions with the greatest contribution in both classifications. In detail, the weights extracted from a certain classifier were first transformed to their absolute values. Then these absolute values were further transformed to their original feature space according to the dimensionality reduction algorithm and normalized as brain region contributions for the ranking process. We selected the top 5% features (this involved a different number of features for the different atlases) with the greatest contribution and further calculated the actual percentage of their contribution.

Finally, to improve the clinical diagnosis, we established an integral model for each classification with the stacking technique (Wolpert, 1992; Ting and Witten, 1999). All models (the combinations of three brain atlases, five classifiers, two cross validation methods, and 3 dimensionality reduction algorithms) generated by the pipeline were selected as level-0 generalizers and the gradient boosting algorithm (Friedman, 2001) was selected as the level-1 generalizer. The best hyperparameter set selected by the pipeline structure mentioned above is also applied for each model in level-0 generalizer, and the hyperparameter set for the level-1 generalizer is selected by a fivefold cross validation. Since it is binary classification on both classifications (SZ or NC in SZ vs NC classification; FESZ or CSZ in FESZ vs NC classification), the input data for the level-1 generalizer is generated as the probability to be classified as one class by all level-0 generalizers in both classifications. Therefore, the dimension of the features was identical to the number of the level-0 generalizers (90 for each classification). The train set and separate test set for level-1 generalizer were generated by fivefold cross validation as detailed described in references (Wolpert, 1992; Ting and Witten, 1999). The performance of the final integral model was tested by the separate test set to guarantee generalization. The hyperparameter set available for the gradient boosting algorithm is shown in Supplementary Table 2.

The whole classification process was realized by the sklearn software package (https://scikit-learn.org/stable/) for machine learning in python code and an in-house software “*NEURO-LEARN*” (https://www.github.com/Raniac/NEURO-LEARN).

## Results

### Overall Classifier Performance

**FIGURE 2:**
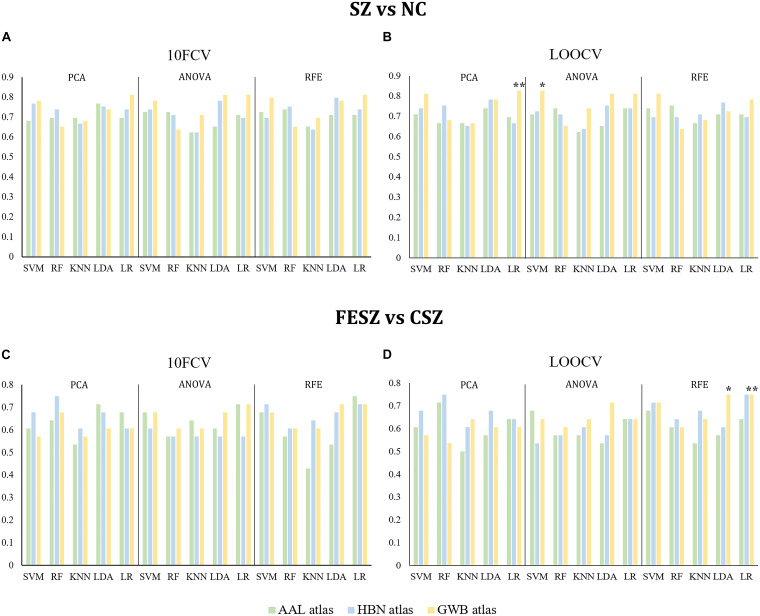
Accuracies of SZ vs NC Classification **(A,B)** and FESZ vs CSZ Classification **(C,D)**. Generally, the accuracies by different combinations of the classifiers and dimensionality reduction algorithms were quite similar. The best combination and the second best combination best combination are highlighted with character * and ** separately on both classifications. 10FCV, 10-fold cross validation; AAL atlas, automated anatomical labeling atlas; ANOVA, analysis of variance; CSZ, chronic schizophrenia; FESZ, first-episode schizophrenia; GWB atlas, groupwise whole-brain atlas; HBN atlas human brainnetome atlas; KNN, K nearest neighbor; LDA, linear discriminant analysis; LOOCV, leave-one-out cross validation; LR, logistic regression; NC, normal control; PCA, principle component analysis; RF, random forest; RFE, recursive feature elimination; SVM, support vector machine; SZ, schizophrenia.

The results of both classifications are shown in Figure 2 and Supplementary Tables 3, 4, and the optimal hyperparameter sets selected for all models are shown in Supplementary Table 5. We selected the models with the highest accuracy and then ranked them by AUC. In the classification between SZ and NC, the best combination was PCA with LR using the GWB atlas with LOOCV (accuracy: 0.83, *P* < 0.05; AUC: 0.89, *P* < 0.05; sensitivity: 0.89; specificity: 0.78; Figure 2B, indicated by ^∗∗^). Moreover, the second-best combination was ANOVA with SVM using the GWB atlas with LOOCV (accuracy: 0.83, *P* < 0.05; AUC: 0.86, *P* < 0.05; sensitivity: 0.71; specificity: 0.90; Figure 2B, indicated by ^∗^). Similarly, in the classification between FESZ and CSZ, the best combination was RFE with LR using the GWB atlas with LOOCV (accuracy: 0.75, *P* < 0.05; AUC: 0.77, *P* < 0.05; sensitivity: 0.80; specificity: 0.69; Figure 2D, indicated by ^∗∗^) and the second-best combination was RFE with LDA using the GWB atlas with LOOCV (accuracy: 0.75, *P* < 0.05; AUC: 0.77, *P* < 0.05; sensitivity: 0.80; specificity: 0.69; Figure 2D, indicated by ^∗^).

Together, it was discovered that: (1) the GWB atlas was the optimal atlas for both classifications and the best results by the HBN atlas (SZ vs NC: RFE-LDA-10FCV; FESZ vs CSZ: RFE-LR-LOOCV) were also comparable; (2) LR and RFE showed a slight advantage over the others, but generally the results with the various combinations of the classifiers and dimensionality reduction algorithms were quite similar; and (3) LOOCV was the best method to identify the best hyperparameter set for both classifications.

### Feature Importance Analysis

**FIGURE 3:**
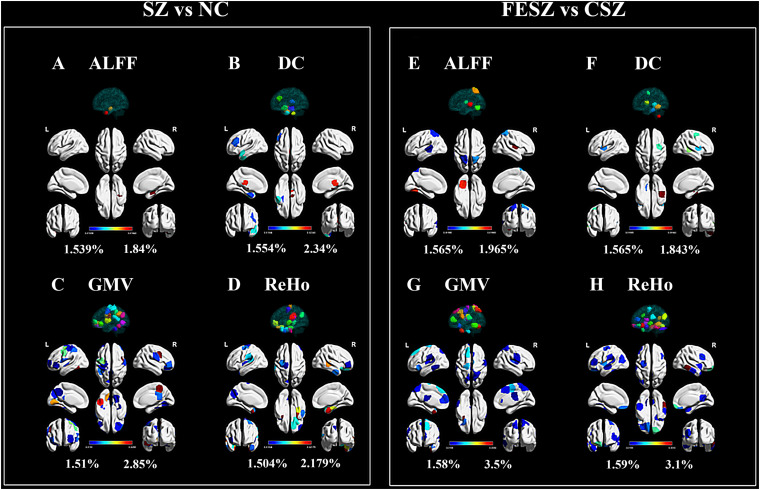
Top 5% ROIs of ALFF **(A,E)**, DC **(B,F)** GMV **(C,G)** and ReHo **(D,H)** with contribution to both classifications. The percentage shown next to the color bar was calculated as the weight of a certain ROI divided by the sum of weights for all 54 ROIs (top 5%) in each group. The color of region projected on the white brain map model referred to the color bar, while the color of the 3D model projected on the transparent brain map model was only applied for ROI distinction, bearing no relevance to the color bar. The figure was generated using BrainNet Viewer (Xia et al., 2013) (http://www.nitrc.org/projects/bnv/). ALFF, amplitude of low frequency fluctuation; CSZ, chronic schizophrenia; DC, degree centrality; FESZ, first-episode schizophrenia; GMV, regional gray matter volume; NC, normal control; ReHo, regional homogeneity; SZ, schizophrenia.

The best combination for two classifications (the combination of the GWB atlas, LR, LOOCV, and PCA for the SZ vs NC classification; the combination of the GWB atlas, LR, LOOCV, and RFE for the FESZ vs CSZ classification) were utilized to generate weights for feature ranking. Based on the methods stated above, the results are shown in Figure 3 and Supplementary Tables 6, 7.

Generally, the GMV and ReHo features equally made the greatest contributions in both classifications that were much more than the contributions of ALFF and DC. In detail, ALFF from the right limbic hippocampus possessed relatively higher weights in the classification between SZ and NC (Figure 3A). For the classification between FESZ vs CSZ, ALFF from the right parietal primary sensory, left occipital primary sensory and somatosensory association cortex made greater contributions (Figure 3E). DC contributed slightly more to the classification than ALFF. The DC with the highest weight came from the right brainstem and right subcortical thalamus for the SZ vs NC classification (Figure 3B), while the highest weights came from the inferior temporal gyrus, middle temporal gyrus and premotor cortex for the FESZ vs CSZ classification (Figure 3F). Features from GMV and ReHo made up approximately 80% of all 54 features selected. The GMV with the greatest contribution was derived from the right premotor cortex, dorsal posterior cingulate cortex, left temporal fusiform cortex, right prefrontal pars opercularis and left orbitofrontal area in the classification between SZ and NC (Figure 3C), while the highest weights were derived from the left temporal pole, left prefrontal visual field, left motor strip, right motor strip and inferior prefrontal gyrus in the classification between FESZ and CSZ (Figure 3G). Meanwhile, the ReHo features with the greatest contributions stemmed from the left orbitofrontal area, left temporal pole, right limbic parahippocampus and right middle temporal gyrus in the classification between SZ and NC (Figure 3D) and the highest weights stemmed from right the temporal fusiform region, right orbitofrontal area, left insula, left cerebellum and left orbitofrontal area in the classification between FESZ and CSZ (Figure 3H).

**FIGURE 4:**
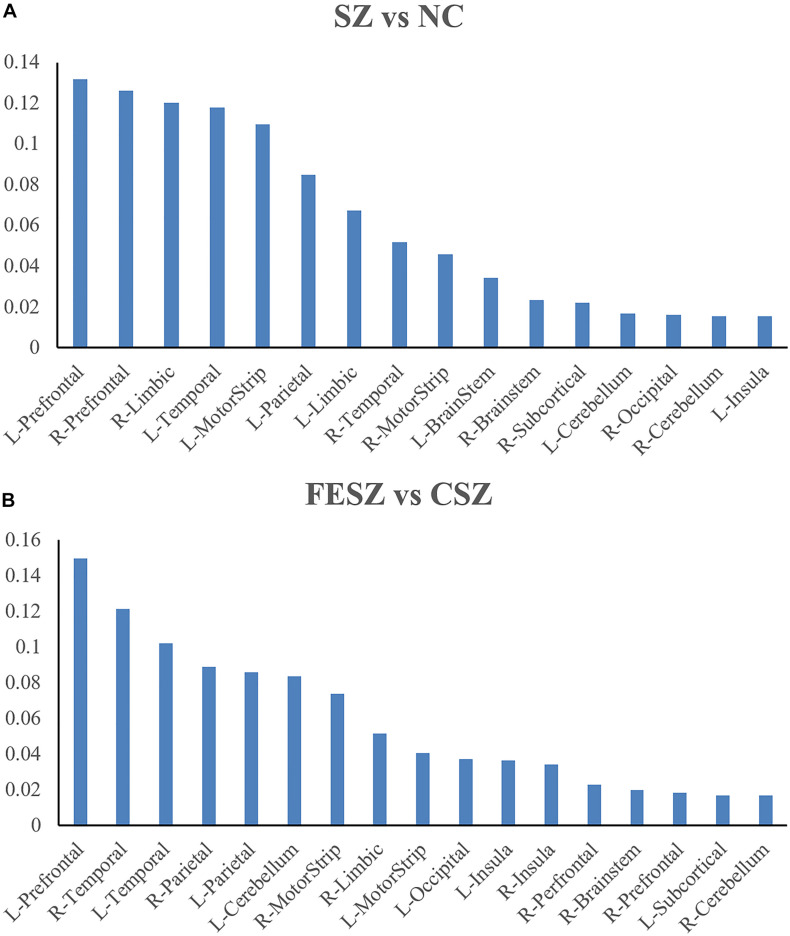
Brain regions with most contribution to SZ vs NC Classification **(A)** and FESZ vs CSZ Classification **(B)**. The percentage shown as the y axis is calculated as the weight of features from a certain brain region divided by the sum of weights of all top 5% features in each classification. The matching between the ROI and the brain region refers to https://bioimagesuiteweb.github.io/webapp/connviewer.html. CSZ, chronic schizophrenia; FESZ, first-episode schizophrenia; L, left; NC, normal control; R, right; SZ, schizophrenia.

Further measurements were also performed for the brain region contribution in both classifications with all four features (ReHo, ALFF, DC, and GMV) considered (Figure 4A for SZ vs NC classification; Figure 4B for FESZ vs CSZ classification). The contribution of a certain brain region was calculated as the sum of contributions of all four features located in that brain region. It is evident that the brain regions that contributed most to the SZ vs NC classification were the left prefrontal cortex, right prefrontal cortex, right limbic cortex, left temporal cortex and left motor strip. Similarly, the brain regions that contributed most to the FESZ vs CSZ classification were the left prefrontal cortex, right temporal cortex and left temporal cortex.

Besides, the best models by the HBN atlas (the combination of the LDA, 10FCV, and RFE for the SZ vs NC classification; the combination of LR, LOOCV, and RFE for the FESZ vs CSZ classification) were also utilized to generate weights for feature ranking. The result shows that GMV and ReHo features have made more contribution than ALFF and DC features to both classifications (in Supplementary Figure 3 and Supplementary Tables 8, 9), which is consistent with the result by GWB atlas. Moreover, the contributory features from HBN atlas were also mainly from frontal cortex and temporal cortex as the same of GWB atlas, indicating the commonality of two atlas on extracting features on discriminative analysis for schizophrenia (in Supplementary Figure 4 and Supplementary Tables 8, 9). On the other hand, the contributory ROIs from HBN atlas were not exactly the same as those from GWB atlas as shown in Supplementary Figure 3. This shows different brain atlases, although with similar number of ROIs, might still result in the different selection of features for the machine learning models.

### Predictive Model Performance

The improvements in the performance with the integral model was evident as shown in Table 2. The accuracy and AUC were significantly increased by the stacking algorithm on the separate test set in both classifications (SZ vs NC: accuracy = 0.88, AUC = 0.92; FESZ vs CSZ: accuracy = 0.86, AUC = 0.80).

**TABLE 2:** Classification performance improvement for integral model.

**Classification group**	**SZ vs NC**		**FESZ vs CSZ**	
**Before/After stacking**	**Before stacking**	**After stacking**	**Before stacking**	**After stacking**
Accuracy	0.83	0.88	0.75	0.86
AUC	0.89	0.92	0.77	0.80

*The accuracy and AUC before stacking were selected from the optimal model for both classifications, respectively. It is clear from the results that the accuracy and AUC were significantly improved by stacking technique to establish the integral model.*

*AUC, the area under receiver operating characteristic curve; CSZ, chronic schizophrenia; FESZ, first episode schizophrenia; NC, normal control.*

## Discussion

In this study, we systematically analyzed classification performances by using multiple brain atlases and multiple machine learning methods with multimodal MRI data. The main findings are as follows: 1) the GWB parcellation with 268 ROIs outperformed the other two brain atlases; (2) the LOOCV was the best method of cross validation to select the best hyperparameter set, but the results with different classifiers and dimensionality reduction algorithms were quite similar; (3) the GMV and ReHo features in the prefrontal and temporal gyri made the greatest contributions in both classifications; and (4) the ensemble learning method substantially improved classification performance.

Generally, the selection of the brain atlas may result in striking differences in performance of the classification of psychiatric diseases (Koikkalainen et al., 2011; Min et al., 2014; Liu J. et al., 2017; Asim et al., 2018; Kalmady et al., 2019). Kalmady et al. (2019) used 14 brain atlases for discriminative analyses of SZ patients and found that the accuracies of the classifications varied significantly across different brain atlases. Similarly, a number of discriminative analyses have also been performed with patients with Alzheimer’s disease (AD) based on multiple brain atlases (Koikkalainen et al., 2011; Min et al., 2014; Asim et al., 2018), in which the features based on all atlases were used to establish the integral model and achieved the best classification performance (Koikkalainen et al., 2011; Min et al., 2014; Asim et al., 2018). Consistent with previous studies, our results also showed discrepancies in the classification performance with different brain atlases. Moreover, our results also suggested the apparent superiority of the GWB atlas, compared with the AAL atlas and the HBN atlas. Previous studies have indicated that the utilization of the GWB atlas has also resulted in satisfactory performances on other discriminative studies, which is consistent with our findings (Valizadeh et al., 2018; de Souza Rodrigues et al., 2019). We speculated that this superiority may derive from the node number and the construction method of the GWB atlas. First, the number of nodes in the GWB atlas is consistent with the range proposed by other studies (Craddock et al., 2012; Van Essen et al., 2012), which enables the brain atlas to provide a more fine-grained scheme than other brain atlases such as the AAL atlas (Finn et al., 2015). Second, the construction of the GWB atlas is based on a groupwise parcellation method, which guaranteed that each node contains voxels with similar resting-state timecourses (Bianciardi et al., 2009; Finn et al., 2015). This could ensure homogeneity within each node and thus better discriminability of the features from the MRI data (Shen et al., 2013). Therefore, these two traits may serve as important criteria for the selection of brain atlases in discriminative analysis of SZ patients.

The selection of machine learning methods has been consequential with regard to the classification process in recent studies (Watanabe et al., 2014; Iqbal et al., 2017; Raymond et al., 2017; Junhua et al., 2018). In this study, we found that the results with different combinations of classifiers and dimensionality reduction algorithms were quite similar, which is consistent with previous studies (Khondoker et al., 2013; Raymond et al., 2017). In detail, although the LR and RFE exhibited a slight advantage over the others, PCA, ANOVA, SVM, and LDA could also be acceptable choices for the classification. Importantly, our results were surprising because most of the classifiers and dimensionality reduction algorithms were mathematically distinct. One of the plausible explanations is the inherent similarities within different classifiers (Hastie et al., 2008). Almost all classifiers are able to generate a hyperplane, which is the best geometrical feature to classify the distributions of the data in multidimensional feature space with unstructured noise (Raymond et al., 2017). Thus, if the multimodal MRI data confirmed to a specific distribution, similar performances would be achieved by different classifiers and dimensionality reduction algorithms. Furthermore, compared with 10-fold cross validation, LOOCV was discovered to be the optimal method for the selection of hyperparameter sets. Taking into consideration that more training data could be applied to classifiers for discriminative analyses with the LOOCV method (Efron, 1983), the advantage of LOOCV can be easily comprehended.

We also found that the GMV and ReHo features better represented the major discrepancies in both classifications than the ALFF and DC features. These results suggested the necessity of using both structural and functional MRI data for the discriminative analysis of SZ patients, which is consistent with previous studies (Dyrba et al., 2015; Zhuang et al., 2019). The abnormalities in brain regions between SZ patients and NCs were primarily in the bilateral prefrontal cortex, right limbic system, left temporal cortex and left motor strip. While the findings on the prefrontal cortex (Janousova et al., 2015; Ou et al., 2015; Zhuang et al., 2019; Webler et al., 2020), limbic system (Shon et al., 2018; Abdolalizadeh et al., 2020; Falakshahi et al., 2020), and temporal cortex (Shu et al., 2012; Ehrlich et al., 2014; Schnack et al., 2014; Koch et al., 2018; Chatterjee et al., 2020) are aligned with previous studies, the discovery of differences in the motor strip has rarely been reported. The motor strip is imperative as the neural hub that participates in perception, action and anticipation in relation to the environment (Schroeder et al., 1994). Thus, abnormalities in the motor strip might elucidate the abnormal conduct behaviors of SZ patients. Moreover, abnormalities were also found in the left prefrontal cortex, right temporal cortex and left temporal cortex between FESZ and CSZ patients, which is consistent with previous findings of the influence of antipsychotic therapies on brain structure and function (Chua et al., 2009; Goghari et al., 2013; Ren et al., 2013; Lesh et al., 2015). Therefore, we hypothesized that the abnormalities in these brain regions might be derived from the side effects of long term antipsychotic drug intake.

In this study, we also established an integrated model with the stacking technique, which remarkably improved the performance of the integral model. Kalmady et al. (2019) applied stacking technique in discriminative analyses of SZ patients and found that the classification performance (accuracy of 87%) outperformed earlier machine learning models. Similarly, Irandoost et al. also found that the stacking technique for classification of individuals with AD and cognitively normal individuals was better than using one classifier and comparable to the state-of-the-art methods (Irandoost and Asadi, 2019). Consistent with previous studies, our results showed apparent improvements in classification performance after the stacking technique was applied (SZ vs NC: accuracy = 0.88, AUC = 0.92; FESZ vs CSZ: accuracy = 0.86, AUC = 0.80). The advantage of the stacking technique may derive from both the diversity of the level-0 generalizers and the diversity of the atlases, which offer the integral model with more information to learn that reduced the variance (Wolpert, 1992; Ting and Witten, 1999).

## Limitations

There were several limitations in this study. First, the cross validation and the separate test set prevented overfitting and guaranteed model performance for generalization to unseen data, but as a consequence, the accuracies in the FESZ vs CSZ classification (86%) and in the SZ vs NC classification (88%) were still not as satisfactory as in other studies (Iqbal et al., 2017; Liu J. et al., 2017). Similarly, the performance of the model was checked with a limited dataset, because only a modicum of examples had been provided for the model to discover the significant discrepancies between the two groups, especially for the FESZ vs CSZ classification with fewer data. Second, all classifiers applied in this study were traditional classifiers. Recent studies have shown satisfactory classification performance by deep learning methods for psychiatric diseases (Zeng et al., 2018; Matsubara et al., 2019). In future studies, we plan to perform systematic estimations using deep learning methods. Third, more brain atlases of different sizes can be included in the studies (Kalmady et al., 2019). Wu et al. (2018) have used a brain atlas with 1,024 ROIs in the discriminative analysis of SZ patients and achieved high classification performance. Thus, we plan to estimate classification performances based on brain atlases with relatively larger sizes in the future. Moreover, numerous researches using other biological data have also found significant discrepancies between schizophrenia patients and normal controls, including gut microbiota data (Li et al., 2020), blood data (Chan et al., 2014), and electroencephalogram data (Alfimova and Uvarova, 2008). Therefore, we also plan to use multi-biological data on the discriminative analysis for further improvement.

## Conclusion

In this study, a systematic analysis of classifications with different combinations of brain atlases, classifiers, cross validation methods and dimensionality reduction algorithms was performed in two classifications (NC vs SZ, FESZ vs CSZ). The performances of the models were analyzed and the weights from the best combination model were used for feature ranking. Further estimation was also performed to provide information indicating the most significant abnormalities in different brain regions. Moreover, an integral model with higher accuracy and AUC was generated with an ensemble learning method. Our findings indicated effects of these factors in constructing effective classifiers for psychiatric diseases and showed that the integrated model has the potential to improve the clinical diagnosis and treatment evaluation of SZ.

## Data Availability Statement

The raw data supporting the conclusions of this article will be made available by the authors, without undue reservation.

## Ethics Statement

The studies involving human participants were reviewed and approved by the Ethics Committees of the Affiliated Brain Hospital of Guangzhou Medical University. The patients/participants provided their written informed consent to participate in this study.

## Author Contributions

All authors contributed toward data analysis, drafting and critically revising the manuscript, gave final approval of the version to be published, and agreed to be accountable for all aspects of the work.

## Conflict of Interest

The authors declare that the research was conducted in the absence of any commercial or financial relationships that could be construed as a potential conflict of interest.

## Publisher’s Note

All claims expressed in this article are solely those of the authors and do not necessarily represent those of their affiliated organizations, or those of the publisher, the editors and the reviewers. Any product that may be evaluated in this article, or claim that may be made by its manufacturer, is not guaranteed or endorsed by the publisher.

## Footnotes

1
http://www.fil.ion.ucl.au.uk/spm/

2
http://rfmri.org/DPARSF
