# Supplementary material for: Effects of Brain Atlases and Machine Learning Methods on the Discrimination of Schizophrenia Patients: A Multimodal MRI Study
Source: Front Neurosci. 2021 Jul 27;15:697168. doi: 10.3389/fnins.2021.697168 (PMC8353157; doi:10.3389/fnins.2021.697168)
Supplement: Supplementary file 1 [file Table_1.docx]

**Supplementary Materials**

**TABLE S1**

Hyperparameter list of classifiers

| Algorithm | | Hyperparameter |
| --- | --- | --- |
| KNN | n_neighbors: [5, 7, 9]; algorithm: ['auto', 'ball_tree', 'kd_tree', 'brute']; p: [1, 2, 3] | |
| SVM | C: [0.001, 0.01, 0.1, 1, 10] | |
| LR | solver: ['newton-cg', 'lbfgs', 'sag', 'saga']； | |
| LDA | solver: ['svd', 'lsqr'] | |
| RF | max_depth: [2, 3, 4, 5]; "min_samples_split": [5]; min_samples_leaf: [5]; criterion: ["gini", "entropy"]; n_estimators: list(range(10, 50, 10)) | |
| GBC | n_estimators: [10, 20, 30, 40, 50]; loss: ['deviance', 'exponential']; criterion: ['friedman_mse', 'mse'] | |

**Note:** n_samples suggests the number of samples in the training set. n_features suggests the number of features of dataset.

**Abbreviation:** GBC, gradient boosting classifier; KNN, K nearest neighbor; LDA, linear discriminant analysis; LR, logistic regression; RF, random forest; SVM, support vector machine.

**TABLE S2**

Hyperparameter list of dimensionality reduction algorithms

| Algorithm | | Hyperparameter |
| --- | --- | --- |
| PCA | n_components: list(range(11, min(n_samples, n_features)-10, min(n_samples, n_features) //20)) | |
| ANOVA | Percentile: list(range(5, 101, 5)_ | |
| RFE | n_features_to_select: list(range(10, n_features, n_features//20)) | |

**Note:** n_samples suggests the number of samples in the training set. n_features suggests the number of features of dataset.

**Abbreviation:** ANOVA, analysis of variance; PCA, principle component analysis; RFE, recursive feature elimination.

**TABLE S3**

Accuracy and AUC results from SZ vs NC classification

| Group | CV | Atlas | DRA | Classifier | Accuracy | AUC |
| --- | --- | --- | --- | --- | --- | --- |
| SZ vs NC | LOOCV | AAL | PCA | SVM | 0.710145 | 0.791812 |
| SZ vs NC | LOOCV | AAL | PCA | RF | 0.666667 | 0.718641 |
| SZ vs NC | LOOCV | AAL | PCA | KNN | 0.666667 | 0.71385 |
| SZ vs NC | LOOCV | AAL | PCA | LDA | 0.73913 | 0.820557 |
| SZ vs NC | LOOCV | AAL | PCA | LR | 0.695652 | 0.75784 |
| SZ vs NC | LOOCV | AAL | ANOVA | SVM | 0.710145 | 0.770906 |
| SZ vs NC | LOOCV | AAL | ANOVA | RF | 0.73913 | 0.748258 |
| SZ vs NC | LOOCV | AAL | ANOVA | KNN | 0.623188 | 0.709495 |
| SZ vs NC | LOOCV | AAL | ANOVA | LDA | 0.652174 | 0.732578 |
| SZ vs NC | LOOCV | AAL | ANOVA | LR | 0.73913 | 0.812718 |
| SZ vs NC | LOOCV | AAL | RFE | SVM | 0.73913 | 0.780923 |
| SZ vs NC | LOOCV | AAL | RFE | RF | 0.753623 | 0.792683 |
| SZ vs NC | LOOCV | AAL | RFE | KNN | 0.666667 | 0.657666 |
| SZ vs NC | LOOCV | AAL | RFE | LDA | 0.710145 | 0.784843 |
| SZ vs NC | LOOCV | AAL | RFE | LR | 0.710145 | 0.797909 |
| SZ vs NC | LOOCV | HBN | PCA | SVM | 0.73913 | 0.830139 |
| SZ vs NC | LOOCV | HBN | PCA | RF | 0.753623 | 0.80662 |
| SZ vs NC | LOOCV | HBN | PCA | KNN | 0.652174 | 0.654617 |
| SZ vs NC | LOOCV | HBN | PCA | LDA | 0.782609 | 0.832753 |
| SZ vs NC | LOOCV | HBN | PCA | LR | 0.666667 | 0.788328 |
| SZ vs NC | LOOCV | HBN | ANOVA | SVM | 0.724638 | 0.812718 |
| SZ vs NC | LOOCV | HBN | ANOVA | RF | 0.710145 | 0.771777 |
| SZ vs NC | LOOCV | HBN | ANOVA | KNN | 0.637681 | 0.66899 |
| SZ vs NC | LOOCV | HBN | ANOVA | LDA | 0.753623 | 0.827526 |
| SZ vs NC | LOOCV | HBN | ANOVA | LR | 0.73913 | 0.808362 |
| SZ vs NC | LOOCV | HBN | RFE | SVM | 0.695652 | 0.81446 |
| SZ vs NC | LOOCV | HBN | RFE | RF | 0.695652 | 0.746516 |
| SZ vs NC | LOOCV | HBN | RFE | KNN | 0.710145 | 0.664634 |
| SZ vs NC | LOOCV | HBN | RFE | LDA | 0.768116 | 0.865854 |
| SZ vs NC | LOOCV | HBN | RFE | LR | 0.695652 | 0.818815 |
| SZ vs NC | LOOCV | GWB | PCA | SVM | 0.811594 | 0.867596 |
| SZ vs NC | LOOCV | GWB | PCA | RF | 0.681159 | 0.828397 |
| SZ vs NC | LOOCV | GWB | PCA | KNN | 0.666667 | 0.726916 |
| SZ vs NC | LOOCV | GWB | PCA | LDA | 0.782609 | 0.877178 |
| SZ vs NC | LOOCV | GWB | PCA | LR | 0.826087 | 0.888502 |
| SZ vs NC | LOOCV | GWB | ANOVA | SVM | 0.826087 | 0.861498 |
| SZ vs NC | LOOCV | GWB | ANOVA | RF | 0.652174 | 0.761324 |
| SZ vs NC | LOOCV | GWB | ANOVA | KNN | 0.73913 | 0.750436 |
| SZ vs NC | LOOCV | GWB | ANOVA | LDA | 0.811594 | 0.883275 |
| SZ vs NC | LOOCV | GWB | ANOVA | LR | 0.811594 | 0.898955 |
| SZ vs NC | LOOCV | GWB | RFE | SVM | 0.811594 | 0.855401 |
| SZ vs NC | LOOCV | GWB | RFE | RF | 0.637681 | 0.759582 |
| SZ vs NC | LOOCV | GWB | RFE | KNN | 0.681159 | 0.752178 |
| SZ vs NC | LOOCV | GWB | RFE | LDA | 0.724638 | 0.797909 |
| SZ vs NC | LOOCV | GWB | RFE | LR | 0.782609 | 0.871951 |
| SZ vs NC | 10FCV | AAL | PCA | SVM | 0.681159 | 0.760453 |
| SZ vs NC | 10FCV | AAL | PCA | RF | 0.695652 | 0.792683 |
| SZ vs NC | 10FCV | AAL | PCA | KNN | 0.695652 | 0.718206 |
| SZ vs NC | 10FCV | AAL | PCA | LDA | 0.768116 | 0.804878 |
| SZ vs NC | 10FCV | AAL | PCA | LR | 0.695652 | 0.807491 |
| SZ vs NC | 10FCV | AAL | ANOVA | SVM | 0.724638 | 0.791376 |
| SZ vs NC | 10FCV | AAL | ANOVA | RF | 0.724638 | 0.753484 |
| SZ vs NC | 10FCV | AAL | ANOVA | KNN | 0.623188 | 0.709495 |
| SZ vs NC | 10FCV | AAL | ANOVA | LDA | 0.652174 | 0.70993 |
| SZ vs NC | 10FCV | AAL | ANOVA | LR | 0.710145 | 0.803136 |
| SZ vs NC | 10FCV | AAL | RFE | SVM | 0.724638 | 0.788328 |
| SZ vs NC | 10FCV | AAL | RFE | RF | 0.73913 | 0.752613 |
| SZ vs NC | 10FCV | AAL | RFE | KNN | 0.652174 | 0.696429 |
| SZ vs NC | 10FCV | AAL | RFE | LDA | 0.710145 | 0.784843 |
| SZ vs NC | 10FCV | AAL | RFE | LR | 0.710145 | 0.805749 |
| SZ vs NC | 10FCV | HBN | PCA | SVM | 0.768116 | 0.829268 |
| SZ vs NC | 10FCV | HBN | PCA | RF | 0.73913 | 0.81446 |
| SZ vs NC | 10FCV | HBN | PCA | KNN | 0.666667 | 0.667683 |
| SZ vs NC | 10FCV | HBN | PCA | LDA | 0.753623 | 0.827526 |
| SZ vs NC | 10FCV | HBN | PCA | LR | 0.73913 | 0.817944 |
| SZ vs NC | 10FCV | HBN | ANOVA | SVM | 0.73913 | 0.811847 |
| SZ vs NC | 10FCV | HBN | ANOVA | RF | 0.710145 | 0.771777 |
| SZ vs NC | 10FCV | HBN | ANOVA | KNN | 0.623188 | 0.684233 |
| SZ vs NC | 10FCV | HBN | ANOVA | LDA | 0.782609 | 0.84669 |
| SZ vs NC | 10FCV | HBN | ANOVA | LR | 0.695652 | 0.794425 |
| SZ vs NC | 10FCV | HBN | RFE | SVM | 0.695652 | 0.808362 |
| SZ vs NC | 10FCV | HBN | RFE | RF | 0.753623 | 0.777875 |
| SZ vs NC | 10FCV | HBN | RFE | KNN | 0.637681 | 0.618902 |
| SZ vs NC | 10FCV | HBN | RFE | LDA | 0.797101 | 0.866725 |
| SZ vs NC | 10FCV | HBN | RFE | LR | 0.73913 | 0.837108 |
| SZ vs NC | 10FCV | GWB | PCA | SVM | 0.782609 | 0.844512 |
| SZ vs NC | 10FCV | GWB | PCA | RF | 0.652174 | 0.763937 |
| SZ vs NC | 10FCV | GWB | PCA | KNN | 0.681159 | 0.693815 |
| SZ vs NC | 10FCV | GWB | PCA | LDA | 0.73913 | 0.866725 |
| SZ vs NC | 10FCV | GWB | PCA | LR | 0.811594 | 0.890244 |
| SZ vs NC | 10FCV | GWB | ANOVA | SVM | 0.782609 | 0.885017 |
| SZ vs NC | 10FCV | GWB | ANOVA | RF | 0.637681 | 0.704704 |
| SZ vs NC | 10FCV | GWB | ANOVA | KNN | 0.710145 | 0.762631 |
| SZ vs NC | 10FCV | GWB | ANOVA | LDA | 0.811594 | 0.870209 |
| SZ vs NC | 10FCV | GWB | ANOVA | LR | 0.811594 | 0.898084 |
| SZ vs NC | 10FCV | GWB | RFE | SVM | 0.797101 | 0.882404 |
| SZ vs NC | 10FCV | GWB | RFE | RF | 0.652174 | 0.789199 |
| SZ vs NC | 10FCV | GWB | RFE | KNN | 0.695652 | 0.749564 |
| SZ vs NC | 10FCV | GWB | RFE | LDA | 0.782609 | 0.864983 |
| SZ vs NC | 10FCV | GWB | RFE | LR | 0.811594 | 0.891986 |

**Abbreviation:** 10FCV, 10-fold cross validation; ANOVA, analysis of variance; AAL atlas, automated anatomical labeling atlas; AUC, area under ROC curve; CV, cross validation; DRA, dimensionality reduction algorithm; GWB atlas, groupwise whole-brain atlas; HBN atlas human brainnetome atlas; KNN, K nearest neighbor; LDA, linear discriminant analysis; LOOCV, leave-one-out cross validation; LR, logistic regression; NC, normal control; PCA, principle component analysis; RF, random forest; RFE, recursive feature elimination; SVM, support vector machine; SZ, schizophrenia.

**TABLE S4**

Accuracy and AUC results from FESZ vs CSZ classification

| Group | CV | Atlas | DRA | Classifier | Accuracy | AUC |
| --- | --- | --- | --- | --- | --- | --- |
| FESZ vs CSZ | LOOCV | AAL | PCA | SVM | 0.607143 | 0.707692 |
| FESZ vs CSZ | LOOCV | AAL | PCA | RF | 0.714286 | 0.723077 |
| FESZ vs CSZ | LOOCV | AAL | PCA | KNN | 0.5 | 0.497436 |
| FESZ vs CSZ | LOOCV | AAL | PCA | LDA | 0.571429 | 0.646154 |
| FESZ vs CSZ | LOOCV | AAL | PCA | LR | 0.642857 | 0.712821 |
| FESZ vs CSZ | LOOCV | AAL | ANOVA | SVM | 0.678571 | 0.723077 |
| FESZ vs CSZ | LOOCV | AAL | ANOVA | RF | 0.571429 | 0.641026 |
| FESZ vs CSZ | LOOCV | AAL | ANOVA | KNN | 0.571429 | 0.661538 |
| FESZ vs CSZ | LOOCV | AAL | ANOVA | LDA | 0.535714 | 0.666667 |
| FESZ vs CSZ | LOOCV | AAL | ANOVA | LR | 0.642857 | 0.712821 |
| FESZ vs CSZ | LOOCV | AAL | RFE | SVM | 0.678571 | 0.676923 |
| FESZ vs CSZ | LOOCV | AAL | RFE | RF | 0.607143 | 0.651282 |
| FESZ vs CSZ | LOOCV | AAL | RFE | KNN | 0.535714 | 0.510256 |
| FESZ vs CSZ | LOOCV | AAL | RFE | LDA | 0.571429 | 0.635897 |
| FESZ vs CSZ | LOOCV | AAL | RFE | LR | 0.642857 | 0.641026 |
| FESZ vs CSZ | LOOCV | HBN | PCA | SVM | 0.678571 | 0.682051 |
| FESZ vs CSZ | LOOCV | HBN | PCA | RF | 0.75 | 0.738462 |
| FESZ vs CSZ | LOOCV | HBN | PCA | KNN | 0.607143 | 0.592308 |
| FESZ vs CSZ | LOOCV | HBN | PCA | LDA | 0.678571 | 0.702564 |
| FESZ vs CSZ | LOOCV | HBN | PCA | LR | 0.642857 | 0.692308 |
| FESZ vs CSZ | LOOCV | HBN | ANOVA | SVM | 0.535714 | 0.569231 |
| FESZ vs CSZ | LOOCV | HBN | ANOVA | RF | 0.571429 | 0.610256 |
| FESZ vs CSZ | LOOCV | HBN | ANOVA | KNN | 0.607143 | 0.569231 |
| FESZ vs CSZ | LOOCV | HBN | ANOVA | LDA | 0.571429 | 0.610256 |
| FESZ vs CSZ | LOOCV | HBN | ANOVA | LR | 0.642857 | 0.589744 |
| FESZ vs CSZ | LOOCV | HBN | RFE | SVM | 0.714286 | 0.8 |
| FESZ vs CSZ | LOOCV | HBN | RFE | RF | 0.642857 | 0.605128 |
| FESZ vs CSZ | LOOCV | HBN | RFE | KNN | 0.678571 | 0.789744 |
| FESZ vs CSZ | LOOCV | HBN | RFE | LDA | 0.607143 | 0.712821 |
| FESZ vs CSZ | LOOCV | HBN | RFE | LR | 0.75 | 0.738462 |
| FESZ vs CSZ | LOOCV | GWB | PCA | SVM | 0.571429 | 0.65641 |
| FESZ vs CSZ | LOOCV | GWB | PCA | RF | 0.535714 | 0.661538 |
| FESZ vs CSZ | LOOCV | GWB | PCA | KNN | 0.642857 | 0.487179 |
| FESZ vs CSZ | LOOCV | GWB | PCA | LDA | 0.607143 | 0.666667 |
| FESZ vs CSZ | LOOCV | GWB | PCA | LR | 0.607143 | 0.661538 |
| FESZ vs CSZ | LOOCV | GWB | ANOVA | SVM | 0.642857 | 0.692308 |
| FESZ vs CSZ | LOOCV | GWB | ANOVA | RF | 0.607143 | 0.569231 |
| FESZ vs CSZ | LOOCV | GWB | ANOVA | KNN | 0.642857 | 0.612821 |
| FESZ vs CSZ | LOOCV | GWB | ANOVA | LDA | 0.714286 | 0.692308 |
| FESZ vs CSZ | LOOCV | GWB | ANOVA | LR | 0.642857 | 0.661538 |
| FESZ vs CSZ | LOOCV | GWB | RFE | SVM | 0.714286 | 0.707692 |
| FESZ vs CSZ | LOOCV | GWB | RFE | RF | 0.607143 | 0.584615 |
| FESZ vs CSZ | LOOCV | GWB | RFE | KNN | 0.642857 | 0.692308 |
| FESZ vs CSZ | LOOCV | GWB | RFE | LDA | 0.75 | 0.764103 |
| FESZ vs CSZ | LOOCV | GWB | RFE | LR | 0.75 | 0.769231 |
| FESZ vs CSZ | 10FCV | AAL | PCA | SVM | 0.607143 | 0.723077 |
| FESZ vs CSZ | 10FCV | AAL | PCA | RF | 0.642857 | 0.717949 |
| FESZ vs CSZ | 10FCV | AAL | PCA | KNN | 0.535714 | 0.523077 |
| FESZ vs CSZ | 10FCV | AAL | PCA | LDA | 0.714286 | 0.692308 |
| FESZ vs CSZ | 10FCV | AAL | PCA | LR | 0.678571 | 0.692308 |
| FESZ vs CSZ | 10FCV | AAL | ANOVA | SVM | 0.678571 | 0.692308 |
| FESZ vs CSZ | 10FCV | AAL | ANOVA | RF | 0.571429 | 0.620513 |
| FESZ vs CSZ | 10FCV | AAL | ANOVA | KNN | 0.642857 | 0.635897 |
| FESZ vs CSZ | 10FCV | AAL | ANOVA | LDA | 0.607143 | 0.687179 |
| FESZ vs CSZ | 10FCV | AAL | ANOVA | LR | 0.714286 | 0.738462 |
| FESZ vs CSZ | 10FCV | AAL | RFE | SVM | 0.678571 | 0.676923 |
| FESZ vs CSZ | 10FCV | AAL | RFE | RF | 0.571429 | 0.646154 |
| FESZ vs CSZ | 10FCV | AAL | RFE | KNN | 0.428571 | 0.328205 |
| FESZ vs CSZ | 10FCV | AAL | RFE | LDA | 0.535714 | 0.615385 |
| FESZ vs CSZ | 10FCV | AAL | RFE | LR | 0.75 | 0.738462 |
| FESZ vs CSZ | 10FCV | HBN | PCA | SVM | 0.678571 | 0.682051 |
| FESZ vs CSZ | 10FCV | HBN | PCA | RF | 0.75 | 0.738462 |
| FESZ vs CSZ | 10FCV | HBN | PCA | KNN | 0.607143 | 0.548718 |
| FESZ vs CSZ | 10FCV | HBN | PCA | LDA | 0.678571 | 0.702564 |
| FESZ vs CSZ | 10FCV | HBN | PCA | LR | 0.607143 | 0.687179 |
| FESZ vs CSZ | 10FCV | HBN | ANOVA | SVM | 0.607143 | 0.671795 |
| FESZ vs CSZ | 10FCV | HBN | ANOVA | RF | 0.571429 | 0.651282 |
| FESZ vs CSZ | 10FCV | HBN | ANOVA | KNN | 0.571429 | 0.520513 |
| FESZ vs CSZ | 10FCV | HBN | ANOVA | LDA | 0.571429 | 0.630769 |
| FESZ vs CSZ | 10FCV | HBN | ANOVA | LR | 0.571429 | 0.564103 |
| FESZ vs CSZ | 10FCV | HBN | RFE | SVM | 0.714286 | 0.8 |
| FESZ vs CSZ | 10FCV | HBN | RFE | RF | 0.607143 | 0.666667 |
| FESZ vs CSZ | 10FCV | HBN | RFE | KNN | 0.642857 | 0.766667 |
| FESZ vs CSZ | 10FCV | HBN | RFE | LDA | 0.678571 | 0.758974 |
| FESZ vs CSZ | 10FCV | HBN | RFE | LR | 0.714286 | 0.835897 |
| FESZ vs CSZ | 10FCV | GWB | PCA | SVM | 0.571429 | 0.671795 |
| FESZ vs CSZ | 10FCV | GWB | PCA | RF | 0.678571 | 0.65641 |
| FESZ vs CSZ | 10FCV | GWB | PCA | KNN | 0.571429 | 0.523077 |
| FESZ vs CSZ | 10FCV | GWB | PCA | LDA | 0.607143 | 0.666667 |
| FESZ vs CSZ | 10FCV | GWB | PCA | LR | 0.607143 | 0.661538 |
| FESZ vs CSZ | 10FCV | GWB | ANOVA | SVM | 0.678571 | 0.692308 |
| FESZ vs CSZ | 10FCV | GWB | ANOVA | RF | 0.607143 | 0.579487 |
| FESZ vs CSZ | 10FCV | GWB | ANOVA | KNN | 0.607143 | 0.607692 |
| FESZ vs CSZ | 10FCV | GWB | ANOVA | LDA | 0.678571 | 0.702564 |
| FESZ vs CSZ | 10FCV | GWB | ANOVA | LR | 0.714286 | 0.769231 |
| FESZ vs CSZ | 10FCV | GWB | RFE | SVM | 0.678571 | 0.712821 |
| FESZ vs CSZ | 10FCV | GWB | RFE | RF | 0.607143 | 0.579487 |
| FESZ vs CSZ | 10FCV | GWB | RFE | KNN | 0.607143 | 0.54359 |
| FESZ vs CSZ | 10FCV | GWB | RFE | LDA | 0.714286 | 0.753846 |
| FESZ vs CSZ | 10FCV | GWB | RFE | LR | 0.714286 | 0.774359 |

**Abbreviation:** 10FCV, 10-fold cross validation; AAL atlas, automated anatomical labeling atlas; ANOVA, analysis of variance; AUC, area under ROC curve; CSZ, chronic schizophrenia; CV, cross validation; DRA, dimensionality reduction algorithm; FESZ, first-episode schizophrenia; GWB atlas, groupwise whole-brain atlas; HBN atlas human brainnetome atlas; KNN, K nearest neighbor; LDA, linear discriminant analysis; LOOCV, leave-one-out cross validation; LR, logistic regression; PCA, principle component analysis; RF, random forest; RFE, recursive feature elimination; SVM, support vector machine.

**TABLE S5**

Optimal hyperparameter selection for all models in both classifications

| Group | CV | Atlas | DRA | Classifier | Hyperparameters |
| --- | --- | --- | --- | --- | --- |
| FESZ vs CSZ | LOOCV | AAL | PCA | SVM | {'PCA__n_components': 21, 'SVM__C': 0.01} |
| FESZ vs CSZ | LOOCV | AAL | PCA | RF | {'PCA__n_components': 21, 'RF__bootstrap': True, 'RF__criterion': 'entropy', 'RF__max_depth': 3, 'RF__min_samples_leaf': 10, 'RF__min_samples_split': 5, 'RF__n_estimators': 50} |
| FESZ vs CSZ | LOOCV | AAL | PCA | KNN | {'KNN__algorithm': 'auto', 'KNN__n_neighbors': 9, 'KNN__p': 2, 'PCA__n_components': 86} |
| FESZ vs CSZ | LOOCV | AAL | PCA | LDA | {'LDA__solver': 'svd', 'PCA__n_components': 46} |
| FESZ vs CSZ | LOOCV | AAL | PCA | LR | {'LR__multi_class': 'ovr', 'LR__solver': 'saga', 'PCA__n_components': 61} |
| FESZ vs CSZ | LOOCV | AAL | ANOVA | SVM | {'ANOVA__percentile': 35, 'SVM__C': 0.01} |
| FESZ vs CSZ | LOOCV | AAL | ANOVA | RF | {'ANOVA__percentile': 25, 'RF__bootstrap': True, 'RF__criterion': 'entropy', 'RF__max_depth': 4, 'RF__min_samples_leaf': 10, 'RF__min_samples_split': 5, 'RF__n_estimators': 50} |
| FESZ vs CSZ | LOOCV | AAL | ANOVA | KNN | {'ANOVA__percentile': 20, 'KNN__algorithm': 'auto', 'KNN__n_neighbors': 7, 'KNN__p': 3} |
| FESZ vs CSZ | LOOCV | AAL | ANOVA | LDA | {'ANOVA__percentile': 95, 'LDA__solver': 'svd'} |
| FESZ vs CSZ | LOOCV | AAL | ANOVA | LR | {'ANOVA__percentile': 35, 'LR__multi_class': 'ovr', 'LR__solver': 'saga'} |
| FESZ vs CSZ | LOOCV | AAL | RFE | SVM | {'RFE__n_features_to_select': 46, 'SVM__C': 0.01} |
| FESZ vs CSZ | LOOCV | AAL | RFE | RF | {'RF__bootstrap': True, 'RF__criterion': 'entropy', 'RF__max_depth': 2, 'RF__min_samples_leaf': 10, 'RF__min_samples_split': 5, 'RF__n_estimators': 50, 'RFE__n_features_to_select': 136} |
| FESZ vs CSZ | LOOCV | AAL | RFE | KNN | {'KNN__algorithm': 'auto', 'KNN__n_neighbors': 9, 'KNN__p': 2, 'RFE__n_features_to_select': 298} |
| FESZ vs CSZ | LOOCV | AAL | RFE | LDA | {'LDA__solver': 'svd', 'RFE__n_features_to_select': 28} |
| FESZ vs CSZ | LOOCV | AAL | RFE | LR | {'LR__multi_class': 'multinomial', 'LR__solver': 'newton-cg', 'RFE__n_features_to_select': 28} |
| FESZ vs CSZ | LOOCV | HBN | PCA | SVM | {'PCA__n_components': 11, 'SVM__C': 0.1} |
| FESZ vs CSZ | LOOCV | HBN | PCA | RF | {'PCA__n_components': 11, 'RF__bootstrap': True, 'RF__criterion': 'entropy', 'RF__max_depth': 2, 'RF__min_samples_leaf': 10, 'RF__min_samples_split': 5, 'RF__n_estimators': 50} |
| FESZ vs CSZ | LOOCV | HBN | PCA | KNN | {'KNN__algorithm': 'auto', 'KNN__n_neighbors': 9, 'KNN__p': 2, 'PCA__n_components': 31} |
| FESZ vs CSZ | LOOCV | HBN | PCA | LDA | {'LDA__solver': 'svd', 'PCA__n_components': 11} |
| FESZ vs CSZ | LOOCV | HBN | PCA | LR | {'LR__multi_class': 'multinomial', 'LR__solver': 'saga', 'PCA__n_components': 11} |
| FESZ vs CSZ | LOOCV | HBN | ANOVA | SVM | {'ANOVA__percentile': 5, 'SVM__C': 0.1} |
| FESZ vs CSZ | LOOCV | HBN | ANOVA | RF | {'ANOVA__percentile': 5, 'RF__bootstrap': True, 'RF__criterion': 'entropy', 'RF__max_depth': 3, 'RF__min_samples_leaf': 10, 'RF__min_samples_split': 5, 'RF__n_estimators': 50} |
| FESZ vs CSZ | LOOCV | HBN | ANOVA | KNN | {'ANOVA__percentile': 5, 'KNN__algorithm': 'auto', 'KNN__n_neighbors': 7, 'KNN__p': 3} |
| FESZ vs CSZ | LOOCV | HBN | ANOVA | LDA | {'ANOVA__percentile': 5, 'LDA__solver': 'svd'} |
| FESZ vs CSZ | LOOCV | HBN | ANOVA | LR | {'ANOVA__percentile': 5, 'LR__multi_class': 'ovr', 'LR__solver': 'newton-cg'} |
| FESZ vs CSZ | LOOCV | HBN | RFE | SVM | {'RFE__n_features_to_select': 206, 'SVM__C': 0.01} |
| FESZ vs CSZ | LOOCV | HBN | RFE | RF | {'RF__bootstrap': True, 'RF__criterion': 'entropy', 'RF__max_depth': 2, 'RF__min_samples_leaf': 10, 'RF__min_samples_split': 5, 'RF__n_estimators': 50, 'RFE__n_features_to_select': 304} |
| FESZ vs CSZ | LOOCV | HBN | RFE | KNN | {'KNN__algorithm': 'auto', 'KNN__n_neighbors': 7, 'KNN__p': 3, 'RFE__n_features_to_select': 59} |
| FESZ vs CSZ | LOOCV | HBN | RFE | LDA | {'LDA__solver': 'svd', 'RFE__n_features_to_select': 549} |
| FESZ vs CSZ | LOOCV | HBN | RFE | LR | {'LR__multi_class': 'ovr', 'LR__solver': 'newton-cg', 'RFE__n_features_to_select': 59} |
| FESZ vs CSZ | LOOCV | GWB | PCA | SVM | {'PCA__n_components': 11, 'SVM__C': 0.01} |
| FESZ vs CSZ | LOOCV | GWB | PCA | RF | {'PCA__n_components': 11, 'RF__bootstrap': True, 'RF__criterion': 'entropy', 'RF__max_depth': 2, 'RF__min_samples_leaf': 10, 'RF__min_samples_split': 5, 'RF__n_estimators': 50} |
| FESZ vs CSZ | LOOCV | GWB | PCA | KNN | {'KNN__algorithm': 'auto', 'KNN__n_neighbors': 9, 'KNN__p': 1, 'PCA__n_components': 16} |
| FESZ vs CSZ | LOOCV | GWB | PCA | LDA | {'LDA__solver': 'lsqr', 'PCA__n_components': 11} |
| FESZ vs CSZ | LOOCV | GWB | PCA | LR | {'LR__multi_class': 'ovr', 'LR__solver': 'sag', 'PCA__n_components': 11} |
| FESZ vs CSZ | LOOCV | GWB | ANOVA | SVM | {'ANOVA__percentile': 20, 'SVM__C': 0.01} |
| FESZ vs CSZ | LOOCV | GWB | ANOVA | RF | {'ANOVA__percentile': 70, 'RF__bootstrap': True, 'RF__criterion': 'entropy', 'RF__max_depth': 3, 'RF__min_samples_leaf': 10, 'RF__min_samples_split': 5, 'RF__n_estimators': 50} |
| FESZ vs CSZ | LOOCV | GWB | ANOVA | KNN | {'ANOVA__percentile': 20, 'KNN__algorithm': 'auto', 'KNN__n_neighbors': 9, 'KNN__p': 3} |
| FESZ vs CSZ | LOOCV | GWB | ANOVA | LDA | {'ANOVA__percentile': 35, 'LDA__solver': 'svd'} |
| FESZ vs CSZ | LOOCV | GWB | ANOVA | LR | {'ANOVA__percentile': 10, 'LR__multi_class': 'multinomial', 'LR__solver': 'newton-cg'} |
| FESZ vs CSZ | LOOCV | GWB | RFE | SVM | {'RFE__n_features_to_select': 646, 'SVM__C': 0.001} |
| FESZ vs CSZ | LOOCV | GWB | RFE | RF | {'RF__bootstrap': True, 'RF__criterion': 'entropy', 'RF__max_depth': 4, 'RF__min_samples_leaf': 10, 'RF__min_samples_split': 5, 'RF__n_estimators': 50, 'RFE__n_features_to_select': 116} |
| FESZ vs CSZ | LOOCV | GWB | RFE | KNN | {'KNN__algorithm': 'auto', 'KNN__n_neighbors': 7, 'KNN__p': 2, 'RFE__n_features_to_select': 63} |
| FESZ vs CSZ | LOOCV | GWB | RFE | LDA | {'LDA__solver': 'svd', 'RFE__n_features_to_select': 699} |
| FESZ vs CSZ | LOOCV | GWB | RFE | LR | {'LR__multi_class': 'ovr', 'LR__solver': 'newton-cg', 'RFE__n_features_to_select': 169} |
| FESZ vs CSZ | 10FCV | AAL | PCA | SVM | {'PCA__n_components': 59, 'SVM__C': 0.01} |
| FESZ vs CSZ | 10FCV | AAL | PCA | RF | {'PCA__n_components': 15, 'RF__bootstrap': True, 'RF__criterion': 'entropy', 'RF__max_depth': 2, 'RF__min_samples_leaf': 10, 'RF__min_samples_split': 5, 'RF__n_estimators': 50} |
| FESZ vs CSZ | 10FCV | AAL | PCA | KNN | {'KNN__algorithm': 'auto', 'KNN__n_neighbors': 7, 'KNN__p': 2, 'PCA__n_components': 79} |
| FESZ vs CSZ | 10FCV | AAL | PCA | LDA | {'LDA__solver': 'svd', 'PCA__n_components': 11} |
| FESZ vs CSZ | 10FCV | AAL | PCA | LR | {'LR__multi_class': 'ovr', 'LR__solver': 'newton-cg', 'PCA__n_components': 11} |
| FESZ vs CSZ | 10FCV | AAL | ANOVA | SVM | {'ANOVA__percentile': 70, 'SVM__C': 0.01} |
| FESZ vs CSZ | 10FCV | AAL | ANOVA | RF | {'ANOVA__percentile': 20, 'RF__bootstrap': True, 'RF__criterion': 'entropy', 'RF__max_depth': 2, 'RF__min_samples_leaf': 10, 'RF__min_samples_split': 5, 'RF__n_estimators': 50} |
| FESZ vs CSZ | 10FCV | AAL | ANOVA | KNN | {'ANOVA__percentile': 45, 'KNN__algorithm': 'auto', 'KNN__n_neighbors': 9, 'KNN__p': 3} |
| FESZ vs CSZ | 10FCV | AAL | ANOVA | LDA | {'ANOVA__percentile': 55, 'LDA__solver': 'svd'} |
| FESZ vs CSZ | 10FCV | AAL | ANOVA | LR | {'ANOVA__percentile': 95, 'LR__multi_class': 'ovr', 'LR__solver': 'saga'} |
| FESZ vs CSZ | 10FCV | AAL | RFE | SVM | {'RFE__n_features_to_select': 46, 'SVM__C': 0.01} |
| FESZ vs CSZ | 10FCV | AAL | RFE | RF | {'RF__bootstrap': True, 'RF__criterion': 'entropy', 'RF__max_depth': 2, 'RF__min_samples_leaf': 10, 'RF__min_samples_split': 5, 'RF__n_estimators': 50, 'RFE__n_features_to_select': 244} |
| FESZ vs CSZ | 10FCV | AAL | RFE | KNN | {'KNN__algorithm': 'auto', 'KNN__n_neighbors': 9, 'KNN__p': 3, 'RFE__n_features_to_select': 10} |
| FESZ vs CSZ | 10FCV | AAL | RFE | LDA | {'LDA__solver': 'svd', 'RFE__n_features_to_select': 352} |
| FESZ vs CSZ | 10FCV | AAL | RFE | LR | {'LR__multi_class': 'ovr', 'LR__solver': 'saga', 'RFE__n_features_to_select': 352} |
| FESZ vs CSZ | 10FCV | HBN | PCA | SVM | {'PCA__n_components': 11, 'SVM__C': 0.1} |
| FESZ vs CSZ | 10FCV | HBN | PCA | RF | {'PCA__n_components': 11, 'RF__bootstrap': True, 'RF__criterion': 'entropy', 'RF__max_depth': 2, 'RF__min_samples_leaf': 10, 'RF__min_samples_split': 5, 'RF__n_estimators': 50} |
| FESZ vs CSZ | 10FCV | HBN | PCA | KNN | {'KNN__algorithm': 'auto', 'KNN__n_neighbors': 9, 'KNN__p': 2, 'PCA__n_components': 19} |
| FESZ vs CSZ | 10FCV | HBN | PCA | LDA | {'LDA__solver': 'svd', 'PCA__n_components': 11} |
| FESZ vs CSZ | 10FCV | HBN | PCA | LR | {'LR__multi_class': 'ovr', 'LR__solver': 'saga', 'PCA__n_components': 11} |
| FESZ vs CSZ | 10FCV | HBN | ANOVA | SVM | {'ANOVA__percentile': 15, 'SVM__C': 0.01} |
| FESZ vs CSZ | 10FCV | HBN | ANOVA | RF | {'ANOVA__percentile': 80, 'RF__bootstrap': True, 'RF__criterion': 'entropy', 'RF__max_depth': 4, 'RF__min_samples_leaf': 10, 'RF__min_samples_split': 5, 'RF__n_estimators': 50} |
| FESZ vs CSZ | 10FCV | HBN | ANOVA | KNN | {'ANOVA__percentile': 5, 'KNN__algorithm': 'auto', 'KNN__n_neighbors': 5, 'KNN__p': 3} |
| FESZ vs CSZ | 10FCV | HBN | ANOVA | LDA | {'ANOVA__percentile': 75, 'LDA__solver': 'svd'} |
| FESZ vs CSZ | 10FCV | HBN | ANOVA | LR | {'ANOVA__percentile': 10, 'LR__multi_class': 'ovr', 'LR__solver': 'newton-cg'} |
| FESZ vs CSZ | 10FCV | HBN | RFE | SVM | {'RFE__n_features_to_select': 206, 'SVM__C': 0.01} |
| FESZ vs CSZ | 10FCV | HBN | RFE | RF | {'RF__bootstrap': True, 'RF__criterion': 'entropy', 'RF__max_depth': 4, 'RF__min_samples_leaf': 10, 'RF__min_samples_split': 5, 'RF__n_estimators': 50, 'RFE__n_features_to_select': 892} |
| FESZ vs CSZ | 10FCV | HBN | RFE | KNN | {'KNN__algorithm': 'auto', 'KNN__n_neighbors': 7, 'KNN__p': 3, 'RFE__n_features_to_select': 108} |
| FESZ vs CSZ | 10FCV | HBN | RFE | LDA | {'LDA__solver': 'lsqr', 'RFE__n_features_to_select': 10} |
| FESZ vs CSZ | 10FCV | HBN | RFE | LR | {'LR__multi_class': 'ovr', 'LR__solver': 'newton-cg', 'RFE__n_features_to_select': 157} |
| FESZ vs CSZ | 10FCV | GWB | PCA | SVM | {'PCA__n_components': 71, 'SVM__C': 0.001} |
| FESZ vs CSZ | 10FCV | GWB | PCA | RF | {'PCA__n_components': 75, 'RF__bootstrap': True, 'RF__criterion': 'entropy', 'RF__max_depth': 3, 'RF__min_samples_leaf': 10, 'RF__min_samples_split': 5, 'RF__n_estimators': 50} |
| FESZ vs CSZ | 10FCV | GWB | PCA | KNN | {'KNN__algorithm': 'auto', 'KNN__n_neighbors': 9, 'KNN__p': 2, 'PCA__n_components': 51} |
| FESZ vs CSZ | 10FCV | GWB | PCA | LDA | {'LDA__solver': 'svd', 'PCA__n_components': 15} |
| FESZ vs CSZ | 10FCV | GWB | PCA | LR | {'LR__multi_class': 'ovr', 'LR__solver': 'newton-cg', 'PCA__n_components': 15} |
| FESZ vs CSZ | 10FCV | GWB | ANOVA | SVM | {'ANOVA__percentile': 45, 'SVM__C': 0.001} |
| FESZ vs CSZ | 10FCV | GWB | ANOVA | RF | {'ANOVA__percentile': 30, 'RF__bootstrap': True, 'RF__criterion': 'entropy', 'RF__max_depth': 2, 'RF__min_samples_leaf': 10, 'RF__min_samples_split': 5, 'RF__n_estimators': 50} |
| FESZ vs CSZ | 10FCV | GWB | ANOVA | KNN | {'ANOVA__percentile': 10, 'KNN__algorithm': 'auto', 'KNN__n_neighbors': 9, 'KNN__p': 3} |
| FESZ vs CSZ | 10FCV | GWB | ANOVA | LDA | {'ANOVA__percentile': 60, 'LDA__solver': 'svd'} |
| FESZ vs CSZ | 10FCV | GWB | ANOVA | LR | {'ANOVA__percentile': 65, 'LR__multi_class': 'ovr', 'LR__solver': 'sag'} |
| FESZ vs CSZ | 10FCV | GWB | RFE | SVM | {'RFE__n_features_to_select': 328, 'SVM__C': 0.001} |
| FESZ vs CSZ | 10FCV | GWB | RFE | RF | {'RF__bootstrap': True, 'RF__criterion': 'entropy', 'RF__max_depth': 2, 'RF__min_samples_leaf': 10, 'RF__min_samples_split': 5, 'RF__n_estimators': 50, 'RFE__n_features_to_select': 752} |
| FESZ vs CSZ | 10FCV | GWB | RFE | KNN | {'KNN__algorithm': 'auto', 'KNN__n_neighbors': 7, 'KNN__p': 2, 'RFE__n_features_to_select': 116} |
| FESZ vs CSZ | 10FCV | GWB | RFE | LDA | {'LDA__solver': 'svd', 'RFE__n_features_to_select': 911} |
| FESZ vs CSZ | 10FCV | GWB | RFE | LR | {'LR__multi_class': 'ovr', 'LR__solver': 'newton-cg', 'RFE__n_features_to_select': 487} |
| SZ vs NC | LOOCV | AAL | PCA | SVM | {'PCA__n_components': 206, 'SVM__C': 0.1} |
| SZ vs NC | LOOCV | AAL | PCA | RF | {'PCA__n_components': 24, 'RF__bootstrap': True, 'RF__criterion': 'entropy', 'RF__max_depth': 5, 'RF__min_samples_leaf': 10, 'RF__min_samples_split': 5, 'RF__n_estimators': 50} |
| SZ vs NC | LOOCV | AAL | PCA | KNN | {'KNN__algorithm': 'auto', 'KNN__n_neighbors': 7, 'KNN__p': 1, 'PCA__n_components': 232} |
| SZ vs NC | LOOCV | AAL | PCA | LDA | {'LDA__solver': 'svd', 'PCA__n_components': 89} |
| SZ vs NC | LOOCV | AAL | PCA | LR | {'LR__multi_class': 'multinomial', 'LR__solver': 'lbfgs', 'PCA__n_components': 115} |
| SZ vs NC | LOOCV | AAL | ANOVA | SVM | {'ANOVA__percentile': 90, 'SVM__C': 0.1} |
| SZ vs NC | LOOCV | AAL | ANOVA | RF | {'ANOVA__percentile': 95, 'RF__bootstrap': True, 'RF__criterion': 'entropy', 'RF__max_depth': 5, 'RF__min_samples_leaf': 10, 'RF__min_samples_split': 5, 'RF__n_estimators': 50} |
| SZ vs NC | LOOCV | AAL | ANOVA | KNN | {'ANOVA__percentile': 85, 'KNN__algorithm': 'auto', 'KNN__n_neighbors': 9, 'KNN__p': 1} |
| SZ vs NC | LOOCV | AAL | ANOVA | LDA | {'ANOVA__percentile': 90, 'LDA__solver': 'svd'} |
| SZ vs NC | LOOCV | AAL | ANOVA | LR | {'ANOVA__percentile': 90, 'LR__multi_class': 'ovr', 'LR__solver': 'sag'} |
| SZ vs NC | LOOCV | AAL | RFE | SVM | {'RFE__n_features_to_select': 136, 'SVM__C': 0.1} |
| SZ vs NC | LOOCV | AAL | RFE | RF | {'RF__bootstrap': True, 'RF__criterion': 'entropy', 'RF__max_depth': 5, 'RF__min_samples_leaf': 10, 'RF__min_samples_split': 5, 'RF__n_estimators': 50, 'RFE__n_features_to_select': 280} |
| SZ vs NC | LOOCV | AAL | RFE | KNN | {'KNN__algorithm': 'auto', 'KNN__n_neighbors': 9, 'KNN__p': 1, 'RFE__n_features_to_select': 100} |
| SZ vs NC | LOOCV | AAL | RFE | LDA | {'LDA__solver': 'svd', 'RFE__n_features_to_select': 136} |
| SZ vs NC | LOOCV | AAL | RFE | LR | {'LR__multi_class': 'ovr', 'LR__solver': 'saga', 'RFE__n_features_to_select': 190} |
| SZ vs NC | LOOCV | HBN | PCA | SVM | {'PCA__n_components': 193, 'SVM__C': 0.01} |
| SZ vs NC | LOOCV | HBN | PCA | RF | {'PCA__n_components': 24, 'RF__bootstrap': True, 'RF__criterion': 'entropy', 'RF__max_depth': 5, 'RF__min_samples_leaf': 10, 'RF__min_samples_split': 5, 'RF__n_estimators': 50} |
| SZ vs NC | LOOCV | HBN | PCA | KNN | {'KNN__algorithm': 'auto', 'KNN__n_neighbors': 9, 'KNN__p': 1, 'PCA__n_components': 258} |
| SZ vs NC | LOOCV | HBN | PCA | LDA | {'LDA__solver': 'svd', 'PCA__n_components': 63} |
| SZ vs NC | LOOCV | HBN | PCA | LR | {'LR__multi_class': 'ovr', 'LR__solver': 'newton-cg', 'PCA__n_components': 115} |
| SZ vs NC | LOOCV | HBN | ANOVA | SVM | {'ANOVA__percentile': 80, 'SVM__C': 0.01} |
| SZ vs NC | LOOCV | HBN | ANOVA | RF | {'ANOVA__percentile': 60, 'RF__bootstrap': True, 'RF__criterion': 'entropy', 'RF__max_depth': 5, 'RF__min_samples_leaf': 10, 'RF__min_samples_split': 5, 'RF__n_estimators': 50} |
| SZ vs NC | LOOCV | HBN | ANOVA | KNN | {'ANOVA__percentile': 70, 'KNN__algorithm': 'auto', 'KNN__n_neighbors': 7, 'KNN__p': 3} |
| SZ vs NC | LOOCV | HBN | ANOVA | LDA | {'ANOVA__percentile': 95, 'LDA__solver': 'svd'} |
| SZ vs NC | LOOCV | HBN | ANOVA | LR | {'ANOVA__percentile': 70, 'LR__multi_class': 'ovr', 'LR__solver': 'sag'} |
| SZ vs NC | LOOCV | HBN | RFE | SVM | {'RFE__n_features_to_select': 353, 'SVM__C': 0.01} |
| SZ vs NC | LOOCV | HBN | RFE | RF | {'RF__bootstrap': True, 'RF__criterion': 'entropy', 'RF__max_depth': 5, 'RF__min_samples_leaf': 10, 'RF__min_samples_split': 5, 'RF__n_estimators': 50, 'RFE__n_features_to_select': 157} |
| SZ vs NC | LOOCV | HBN | RFE | KNN | {'KNN__algorithm': 'auto', 'KNN__n_neighbors': 7, 'KNN__p': 1, 'RFE__n_features_to_select': 157} |
| SZ vs NC | LOOCV | HBN | RFE | LDA | {'LDA__solver': 'svd', 'RFE__n_features_to_select': 647} |
| SZ vs NC | LOOCV | HBN | RFE | LR | {'LR__multi_class': 'multinomial', 'LR__solver': 'newton-cg', 'RFE__n_features_to_select': 353} |
| SZ vs NC | LOOCV | GWB | PCA | SVM | {'PCA__n_components': 115, 'SVM__C': 0.01} |
| SZ vs NC | LOOCV | GWB | PCA | RF | {'PCA__n_components': 89, 'RF__bootstrap': True, 'RF__criterion': 'entropy', 'RF__max_depth': 5, 'RF__min_samples_leaf': 10, 'RF__min_samples_split': 5, 'RF__n_estimators': 50} |
| SZ vs NC | LOOCV | GWB | PCA | KNN | {'KNN__algorithm': 'auto', 'KNN__n_neighbors': 9, 'KNN__p': 1, 'PCA__n_components': 206} |
| SZ vs NC | LOOCV | GWB | PCA | LDA | {'LDA__solver': 'svd', 'PCA__n_components': 102} |
| SZ vs NC | LOOCV | GWB | PCA | LR | {'LR__multi_class': 'multinomial', 'LR__solver': 'sag', 'PCA__n_components': 219} |
| SZ vs NC | LOOCV | GWB | ANOVA | SVM | {'ANOVA__percentile': 100, 'SVM__C': 0.001} |
| SZ vs NC | LOOCV | GWB | ANOVA | RF | {'ANOVA__percentile': 35, 'RF__bootstrap': True, 'RF__criterion': 'entropy', 'RF__max_depth': 5, 'RF__min_samples_leaf': 10, 'RF__min_samples_split': 5, 'RF__n_estimators': 50} |
| SZ vs NC | LOOCV | GWB | ANOVA | KNN | {'ANOVA__percentile': 65, 'KNN__algorithm': 'auto', 'KNN__n_neighbors': 9, 'KNN__p': 2} |
| SZ vs NC | LOOCV | GWB | ANOVA | LDA | {'ANOVA__percentile': 90, 'LDA__solver': 'svd'} |
| SZ vs NC | LOOCV | GWB | ANOVA | LR | {'ANOVA__percentile': 90, 'LR__multi_class': 'ovr', 'LR__solver': 'sag'} |
| SZ vs NC | LOOCV | GWB | RFE | SVM | {'RFE__n_features_to_select': 169, 'SVM__C': 0.01} |
| SZ vs NC | LOOCV | GWB | RFE | RF | {'RF__bootstrap': True, 'RF__criterion': 'entropy', 'RF__max_depth': 5, 'RF__min_samples_leaf': 10, 'RF__min_samples_split': 5, 'RF__n_estimators': 50, 'RFE__n_features_to_select': 328} |
| SZ vs NC | LOOCV | GWB | RFE | KNN | {'KNN__algorithm': 'auto', 'KNN__n_neighbors': 5, 'KNN__p': 1, 'RFE__n_features_to_select': 1070} |
| SZ vs NC | LOOCV | GWB | RFE | LDA | {'LDA__solver': 'svd', 'RFE__n_features_to_select': 593} |
| SZ vs NC | LOOCV | GWB | RFE | LR | {'LR__multi_class': 'multinomial', 'LR__solver': 'sag', 'RFE__n_features_to_select': 381} |
| SZ vs NC | 10FCV | AAL | PCA | SVM | {'PCA__n_components': 155, 'SVM__C': 1} |
| SZ vs NC | 10FCV | AAL | PCA | RF | {'PCA__n_components': 47, 'RF__bootstrap': True, 'RF__criterion': 'entropy', 'RF__max_depth': 5, 'RF__min_samples_leaf': 10, 'RF__min_samples_split': 5, 'RF__n_estimators': 50} |
| SZ vs NC | 10FCV | AAL | PCA | KNN | {'KNN__algorithm': 'auto', 'KNN__n_neighbors': 9, 'KNN__p': 1, 'PCA__n_components': 215} |
| SZ vs NC | 10FCV | AAL | PCA | LDA | {'LDA__solver': 'svd', 'PCA__n_components': 131} |
| SZ vs NC | 10FCV | AAL | PCA | LR | {'LR__multi_class': 'multinomial', 'LR__solver': 'newton-cg', 'PCA__n_components': 215} |
| SZ vs NC | 10FCV | AAL | ANOVA | SVM | {'ANOVA__percentile': 95, 'SVM__C': 0.1} |
| SZ vs NC | 10FCV | AAL | ANOVA | RF | {'ANOVA__percentile': 80, 'RF__bootstrap': True, 'RF__criterion': 'entropy', 'RF__max_depth': 5, 'RF__min_samples_leaf': 10, 'RF__min_samples_split': 5, 'RF__n_estimators': 50} |
| SZ vs NC | 10FCV | AAL | ANOVA | KNN | {'ANOVA__percentile': 85, 'KNN__algorithm': 'auto', 'KNN__n_neighbors': 9, 'KNN__p': 1} |
| SZ vs NC | 10FCV | AAL | ANOVA | LDA | {'ANOVA__percentile': 100, 'LDA__solver': 'svd'} |
| SZ vs NC | 10FCV | AAL | ANOVA | LR | {'ANOVA__percentile': 100, 'LR__multi_class': 'multinomial', 'LR__solver': 'lbfgs'} |
| SZ vs NC | 10FCV | AAL | RFE | SVM | {'RFE__n_features_to_select': 352, 'SVM__C': 0.1} |
| SZ vs NC | 10FCV | AAL | RFE | RF | {'RF__bootstrap': True, 'RF__criterion': 'entropy', 'RF__max_depth': 5, 'RF__min_samples_leaf': 10, 'RF__min_samples_split': 5, 'RF__n_estimators': 50, 'RFE__n_features_to_select': 352} |
| SZ vs NC | 10FCV | AAL | RFE | KNN | {'KNN__algorithm': 'auto', 'KNN__n_neighbors': 9, 'KNN__p': 1, 'RFE__n_features_to_select': 154} |
| SZ vs NC | 10FCV | AAL | RFE | LDA | {'LDA__solver': 'svd', 'RFE__n_features_to_select': 136} |
| SZ vs NC | 10FCV | AAL | RFE | LR | {'LR__multi_class': 'multinomial', 'LR__solver': 'newton-cg', 'RFE__n_features_to_select': 244} |
| SZ vs NC | 10FCV | HBN | PCA | SVM | {'PCA__n_components': 167, 'SVM__C': 0.01} |
| SZ vs NC | 10FCV | HBN | PCA | RF | {'PCA__n_components': 59, 'RF__bootstrap': True, 'RF__criterion': 'entropy', 'RF__max_depth': 5, 'RF__min_samples_leaf': 10, 'RF__min_samples_split': 5, 'RF__n_estimators': 50} |
| SZ vs NC | 10FCV | HBN | PCA | KNN | {'KNN__algorithm': 'auto', 'KNN__n_neighbors': 9, 'KNN__p': 1, 'PCA__n_components': 83} |
| SZ vs NC | 10FCV | HBN | PCA | LDA | {'LDA__solver': 'svd', 'PCA__n_components': 59} |
| SZ vs NC | 10FCV | HBN | PCA | LR | {'LR__multi_class': 'multinomial', 'LR__solver': 'lbfgs', 'PCA__n_components': 167} |
| SZ vs NC | 10FCV | HBN | ANOVA | SVM | {'ANOVA__percentile': 75, 'SVM__C': 0.01} |
| SZ vs NC | 10FCV | HBN | ANOVA | RF | {'ANOVA__percentile': 60, 'RF__bootstrap': True, 'RF__criterion': 'entropy', 'RF__max_depth': 5, 'RF__min_samples_leaf': 10, 'RF__min_samples_split': 5, 'RF__n_estimators': 50} |
| SZ vs NC | 10FCV | HBN | ANOVA | KNN | {'ANOVA__percentile': 65, 'KNN__algorithm': 'auto', 'KNN__n_neighbors': 7, 'KNN__p': 3} |
| SZ vs NC | 10FCV | HBN | ANOVA | LDA | {'ANOVA__percentile': 100, 'LDA__solver': 'svd'} |
| SZ vs NC | 10FCV | HBN | ANOVA | LR | {'ANOVA__percentile': 70, 'LR__multi_class': 'multinomial', 'LR__solver': 'newton-cg'} |
| SZ vs NC | 10FCV | HBN | RFE | SVM | {'RFE__n_features_to_select': 402, 'SVM__C': 0.1} |
| SZ vs NC | 10FCV | HBN | RFE | RF | {'RF__bootstrap': True, 'RF__criterion': 'entropy', 'RF__max_depth': 5, 'RF__min_samples_leaf': 10, 'RF__min_samples_split': 5, 'RF__n_estimators': 50, 'RFE__n_features_to_select': 108} |
| SZ vs NC | 10FCV | HBN | RFE | KNN | {'KNN__algorithm': 'auto', 'KNN__n_neighbors': 9, 'KNN__p': 3, 'RFE__n_features_to_select': 108} |
| SZ vs NC | 10FCV | HBN | RFE | LDA | {'LDA__solver': 'svd', 'RFE__n_features_to_select': 892} |
| SZ vs NC | 10FCV | HBN | RFE | LR | {'LR__multi_class': 'ovr', 'LR__solver': 'saga', 'RFE__n_features_to_select': 696} |
| SZ vs NC | 10FCV | GWB | PCA | SVM | {'PCA__n_components': 59, 'SVM__C': 0.1} |
| SZ vs NC | 10FCV | GWB | PCA | RF | {'PCA__n_components': 11, 'RF__bootstrap': True, 'RF__criterion': 'entropy', 'RF__max_depth': 4, 'RF__min_samples_leaf': 10, 'RF__min_samples_split': 5, 'RF__n_estimators': 50} |
| SZ vs NC | 10FCV | GWB | PCA | KNN | {'KNN__algorithm': 'auto', 'KNN__n_neighbors': 9, 'KNN__p': 3, 'PCA__n_components': 95} |
| SZ vs NC | 10FCV | GWB | PCA | LDA | {'LDA__solver': 'svd', 'PCA__n_components': 95} |
| SZ vs NC | 10FCV | GWB | PCA | LR | {'LR__multi_class': 'multinomial', 'LR__solver': 'sag', 'PCA__n_components': 191} |
| SZ vs NC | 10FCV | GWB | ANOVA | SVM | {'ANOVA__percentile': 100, 'SVM__C': 0.01} |
| SZ vs NC | 10FCV | GWB | ANOVA | RF | {'ANOVA__percentile': 100, 'RF__bootstrap': True, 'RF__criterion': 'entropy', 'RF__max_depth': 5, 'RF__min_samples_leaf': 10, 'RF__min_samples_split': 5, 'RF__n_estimators': 50} |
| SZ vs NC | 10FCV | GWB | ANOVA | KNN | {'ANOVA__percentile': 70, 'KNN__algorithm': 'auto', 'KNN__n_neighbors': 9, 'KNN__p': 2} |
| SZ vs NC | 10FCV | GWB | ANOVA | LDA | {'ANOVA__percentile': 85, 'LDA__solver': 'svd'} |
| SZ vs NC | 10FCV | GWB | ANOVA | LR | {'ANOVA__percentile': 95, 'LR__multi_class': 'multinomial', 'LR__solver': 'sag'} |
| SZ vs NC | 10FCV | GWB | RFE | SVM | {'RFE__n_features_to_select': 699, 'SVM__C': 0.01} |
| SZ vs NC | 10FCV | GWB | RFE | RF | {'RF__bootstrap': True, 'RF__criterion': 'entropy', 'RF__max_depth': 5, 'RF__min_samples_leaf': 10, 'RF__min_samples_split': 5, 'RF__n_estimators': 50, 'RFE__n_features_to_select': 434} |
| SZ vs NC | 10FCV | GWB | RFE | KNN | {'KNN__algorithm': 'auto', 'KNN__n_neighbors': 9, 'KNN__p': 1, 'RFE__n_features_to_select': 699} |
| SZ vs NC | 10FCV | GWB | RFE | LDA | {'LDA__solver': 'svd', 'RFE__n_features_to_select': 858} |
| SZ vs NC | 10FCV | GWB | RFE | LR | {'LR__multi_class': 'multinomial', 'LR__solver': 'sag', 'RFE__n_features_to_select': 1070} |

**Abbreviation:** 10FCV, 10-fold cross validation; AAL atlas, automated anatomical labeling atlas; ANOVA, analysis of variance; AUC, area under ROC curve; CSZ, chronic schizophrenia; CV, cross validation; DRA, dimensionality reduction algorithm; FESZ, first-episode schizophrenia; GWB atlas, groupwise whole-brain atlas; HBN atlas human brainnetome atlas; KNN, K nearest neighbor; LDA, linear discriminant analysis; LOOCV, leave-one-out cross validation; LR, logistic regression; NC, normal control; PCA, principle component analysis; RF, random forest; RFE, recursive feature elimination; SVM, support vector machine; SZ, schizophrenia.

**TABLE S6**

Feature ranking for SZ vs NC classification

| Feature | Node | Brain Side | Region | Percentage |
| --- | --- | --- | --- | --- |
| GMV | 31 | Right | MotorStrip | 2.85% |
| GMV | 91 | Right | Limbic | 2.80% |
| GMV | 200 | Left | Temporal | 2.69% |
| GMV | 211 | Right | Prefrontal | 2.46% |
| ReHo | 136 | Left | Prefrontal | 2.41% |
| DC | 132 | Right | Brainstem | 2.34% |
| DC | 127 | Right | Subcortical | 2.21% |
| ReHo | 134 | Left | Prefrontal | 2.20% |
| GMV | 166 | Left | MotorStrip | 2.19% |
| ReHo | 189 | Left | Temporal | 2.18% |
| GMV | 165 | Left | MotorStrip | 2.15% |
| ReHo | 97 | Right | Limbic | 2.12% |
| ReHo | 54 | Right | Temporal | 2.01% |
| GMV | 217 | Left | Limbic | 1.97% |
| ReHo | 96 | Right | Limbic | 1.90% |
| DC | 194 | Left | Temporal | 1.87% |
| DC | 188 | Left | Temporal | 1.86% |
| ReHo | 151 | Left | Prefrontal | 1.86% |
| ALFF | 94 | Right | Limbic | 1.84% |
| GMV | 179 | Left | Parietal | 1.82% |
| GMV | 85 | Right | Limbic | 1.82% |
| ReHo | 143 | Left | Prefrontal | 1.82% |
| GMV | 163 | Left | MotorStrip | 1.81% |
| GMV | 8 | Right | Prefrontal | 1.80% |
| ReHo | 18 | Right | Prefrontal | 1.78% |
| ReHo | 4 | Right | Prefrontal | 1.77% |
| GMV | 23 | Right | MotorStrip | 1.74% |
| ReHo | 181 | Left | Parietal | 1.73% |
| DC | 147 | Left | Prefrontal | 1.73% |
| DC | 267 | Left | BrainStem | 1.72% |
| ReHo | 268 | Left | BrainStem | 1.71% |
| DC | 235 | Left | Limbic | 1.71% |
| GMV | 176 | Left | Parietal | 1.70% |
| GMV | 17 | Right | Prefrontal | 1.69% |
| ReHo | 180 | Left | Parietal | 1.69% |
| GMV | 245 | Left | Cerebellum | 1.68% |
| ReHo | 166 | Left | MotorStrip | 1.68% |
| ReHo | 56 | Right | Temporal | 1.67% |
| ReHo | 186 | Left | Temporal | 1.66% |
| GMV | 72 | Right | Occipital | 1.63% |
| GMV | 167 | Left | MotorStrip | 1.60% |
| GMV | 142 | Left | Prefrontal | 1.60% |
| GMV | 151 | Left | Prefrontal | 1.56% |
| ReHo | 10 | Right | Prefrontal | 1.56% |
| ReHo | 176 | Left | Parietal | 1.55% |
| DC | 99 | Right | Limbic | 1.55% |
| GMV | 119 | Right | Cerebellum | 1.55% |
| GMV | 225 | Left | Limbic | 1.55% |
| ReHo | 170 | Left | Insula | 1.55% |
| ReHo | 8 | Right | Prefrontal | 1.55% |
| ReHo | 160 | Left | MotorStrip | 1.55% |
| ALFF | 185 | Left | Temporal | 1.54% |
| GMV | 66 | Right | Temporal | 1.51% |
| ReHo | 223 | Left | Limbic | 1.50% |

**Note:** Node number is derived from parcellation in GWB atlas and percentage is calculated as the weight of a certain feature divided by the sum of weights for all 54 features.

**Abbreviation:** ALFF, amplitude of low frequency fluctuation; DC, degree centrality; GMV, gray matter volume; GWB atlas, groupwise whole-brain atlas; NC, normal control; ReHo, regional homogeneity; SZ, schizophrenia.

**TABLE S7**

Feature ranking for FESZ vs CSZ classification

| Feature | Node | Brain Side | Region | Percentage |
| --- | --- | --- | --- | --- |
| GMV | 189 | Left | Temporal | 3.50% |
| ReHo | 70 | Right | Temporal | 3.10% |
| GMV | 148 | Left | Prefrontal | 2.29% |
| ReHo | 1 | Right | Perfrontal | 2.28% |
| GMV | 160 | Left | MotorStrip | 2.19% |
| GMV | 25 | Right | MotorStrip | 2.17% |
| GMV | 153 | Left | Prefrontal | 2.05% |
| ReHo | 168 | Left | Insula | 2.00% |
| ReHo | 241 | Left | Cerebellum | 1.98% |
| GMV | 131 | Right | Brainstem | 1.98% |
| ALFF | 40 | Right | Parietal | 1.97% |
| GMV | 62 | Right | Temporal | 1.96% |
| ReHo | 137 | Left | Prefrontal | 1.95% |
| ALFF | 207 | Left | Occipital | 1.90% |
| GMV | 140 | Left | Prefrontal | 1.89% |
| ReHo | 166 | Left | MotorStrip | 1.86% |
| ReHo | 152 | Left | Prefrontal | 1.86% |
| ReHo | 22 | Right | Prefrontal | 1.85% |
| DC | 59 | Right | Temporal | 1.84% |
| ReHo | 203 | Left | Occipital | 1.83% |
| GMV | 44 | Right | Parietal | 1.83% |
| GMV | 28 | Right | MotorStrip | 1.83% |
| GMV | 178 | Left | Parietal | 1.82% |
| ReHo | 86 | Right | Limbic | 1.80% |
| ReHo | 55 | Right | Temporal | 1.77% |
| ReHo | 201 | Left | Temporal | 1.77% |
| ReHo | 34 | Right | Insula | 1.76% |
| ReHo | 92 | Right | Limbic | 1.76% |
| ReHo | 154 | Left | Prefrontal | 1.75% |
| GMV | 41 | Right | Parietal | 1.75% |
| ReHo | 181 | Left | Parietal | 1.75% |
| GMV | 173 | Left | Parietal | 1.74% |
| ReHo | 52 | Right | Temporal | 1.73% |
| ReHo | 197 | Left | Temporal | 1.72% |
| DC | 54 | Right | Temporal | 1.71% |
| GMV | 23 | Right | MotorStrip | 1.70% |
| DC | 32 | Right | MotorStrip | 1.69% |
| GMV | 264 | Left | Subcortical | 1.69% |
| ReHo | 248 | Right | Cerebellum | 1.68% |
| ALFF | 41 | Right | Parietal | 1.68% |
| GMV | 40 | Right | Parietal | 1.66% |
| DC | 35 | Right | Insula | 1.65% |
| GMV | 181 | Left | Parietal | 1.65% |
| GMV | 199 | Left | Temporal | 1.65% |
| DC | 168 | Left | Insula | 1.64% |
| DC | 244 | Left | Cerebellum | 1.63% |
| ALFF | 175 | Left | Parietal | 1.63% |
| ReHo | 244 | Left | Cerebellum | 1.61% |
| GMV | 88 | Right | Limbic | 1.61% |
| ReHo | 153 | Left | Prefrontal | 1.59% |
| ALFF | 191 | Left | Temporal | 1.58% |
| GMV | 157 | Left | Prefrontal | 1.58% |
| GMV | 248 | Left | Cerebellum | 1.58% |
| DC | 239 | Left | Cerebellum | 1.57% |

**Note:** Node number is derived from parcellation in GWB atlas and percentage is calculated as the weight of a certain feature divided by the sum of weights for all 54 features.

**Abbreviation:** ALFF, amplitude of low frequency fluctuation; CSZ, chronic schizophrenia; DC, degree centrality; FESZ, first-episode schizophrenia; GMV, gray matter volume; GWB atlas, groupwise whole-brain atlas; ReHo, regional homogeneity.


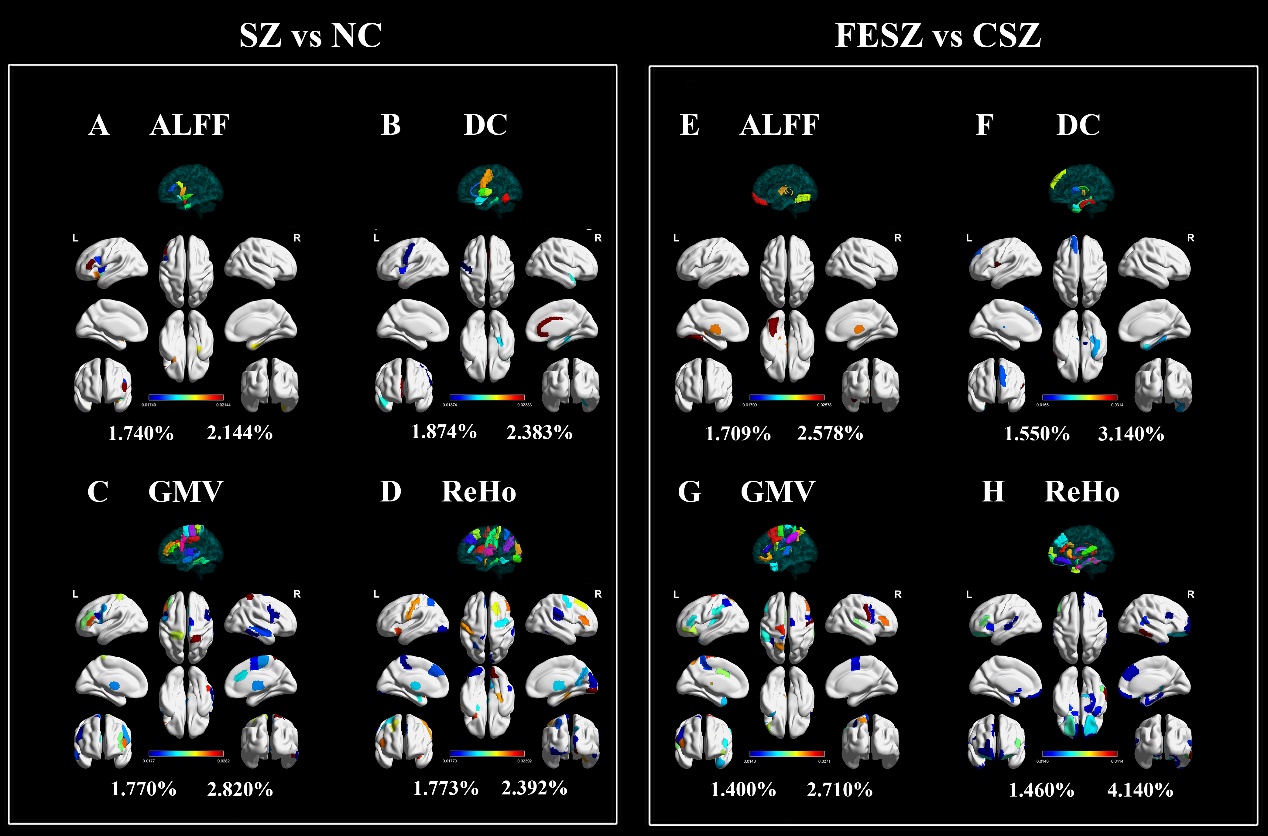


**FIGURE S1.** Top 5% ROIs of ALFF (A, E), DC (B, F) GMV (C, G) and ReHo (D, H) from HBN atlas with contribution to both classifications.

**Note:** The percentage shown next to the color bar was calculated as the weight of a certain ROI divided by the sum of weights for all 49 ROIs (top 5%) in each group. The color of region projected on the white brain map model referred to the color bar, while the color of the 3D model projected on the transparent brain map model was only applied for ROI distinction, bearing no relevance to the color bar. The figure was generated using BrainNet Viewer (http://www.nitrc.org/projects/bnv/).

**Abbreviation:** ALFF, amplitude of low frequency fluctuation; CSZ, chronic schizophrenia; DC, degree centrality; FESZ, first-episode schizophrenia; GMV, regional gray matter volume; HBN atlas, human brainnetome atlas; NC, normal control; ReHo, regional homogeneity; SZ, schizophrenia.


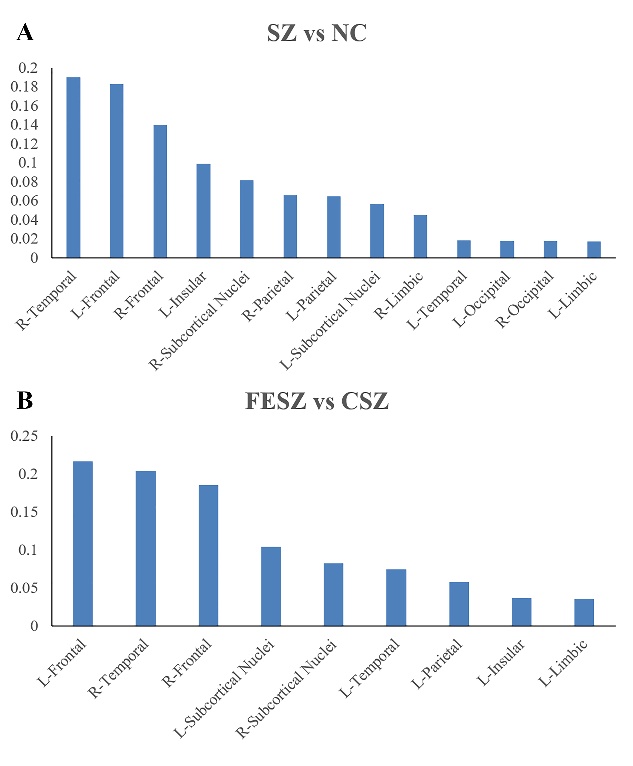


**FIGURE S2.** Brain regions with most contribution to SZ vs NC Classification (A) and FESZ vs CSZ Classification (B) with HBA atlas.

**Note:** The percentage shown as the y axis is calculated as the weight of features from a certain brain region divided by the sum of weights of all top 5% features in each classification. The matching between the ROI and the brain region refers to http://atlas.brainnetome.org/.

**Abbreviation:** CSZ, chronic schizophrenia; FESZ, first-episode schizophrenia; L, left; NC, normal control; R, right; SZ, schizophrenia.

**TABLE S8**

Feature ranking for SZ vs NC classification with HBN atlas

| Feature | Node | Brain Side | Region | Percentage |
| --- | --- | --- | --- | --- |
| GMV | 132 | Right | Parietal | 2.82% |
| GMV | 92 | Right | Temporal | 2.67% |
| GMV | 31 | Left | Frontal | 2.59% |
| ReHo | 190 | Right | Temporal | 2.39% |
| DC | 178 | Right | Limbic | 2.38% |
| GMV | 161 | Left | Parietal | 2.37% |
| ReHo | 244 | Right | Subcortical Nuclei | 2.36% |
| GMV | 21 | Left | Frontal | 2.30% |
| ReHo | 165 | Left | Insular | 2.28% |
| ReHo | 32 | Right | Frontal | 2.25% |
| ReHo | 155 | Left | Parietal | 2.22% |
| ReHo | 114 | Right | Temporal | 2.21% |
| GMV | 180 | Right | Limbic | 2.16% |
| ReHo | 4 | Right | Frontal | 2.15% |
| ALFF | 31 | Left | Frontal | 2.14% |
| DC | 78 | Right | Temporal | 2.08% |
| GMV | 63 | Left | Frontal | 2.07% |
| GMV | 68 | Right | Frontal | 2.07% |
| DC | 114 | Right | Temporal | 2.05% |
| GMV | 238 | Right | Subcortical Nuclei | 2.05% |
| ALFF | 165 | Left | Insular | 2.04% |
| ReHo | 213 | Left | Subcortical Nuclei | 2.02% |
| ReHo | 56 | Right | Frontal | 2.00% |
| ALFF | 110 | Right | Temporal | 1.99% |
| ReHo | 238 | Right | Subcortical Nuclei | 1.99% |
| GMV | 88 | Right | Temporal | 1.97% |
| ReHo | 152 | Right | Parietal | 1.96% |
| DC | 173 | Left | Insular | 1.94% |
| DC | 229 | Left | Subcortical Nuclei | 1.93% |
| ALFF | 169 | Left | Insular | 1.92% |
| GMV | 10 | Right | Frontal | 1.90% |
| ReHo | 11 | Left | Frontal | 1.90% |
| ReHo | 133 | Left | Parietal | 1.90% |
| DC | 53 | Left | Frontal | 1.87% |
| DC | 91 | Left | Temporal | 1.87% |
| ReHo | 92 | Right | Temporal | 1.85% |
| ReHo | 146 | Right | Parietal | 1.82% |
| ReHo | 205 | Left | Occipital | 1.82% |
| ReHo | 65 | Left | Frontal | 1.81% |
| ReHo | 194 | Right | Occipital | 1.81% |
| ALFF | 29 | Left | Frontal | 1.81% |
| GMV | 64 | Right | Frontal | 1.81% |
| GMV | 100 | Right | Temporal | 1.80% |
| GMV | 29 | Left | Frontal | 1.80% |
| GMV | 30 | Right | Frontal | 1.79% |
| ReHo | 228 | Right | Subcortical Nuclei | 1.77% |
| GMV | 185 | Left | Limbic | 1.77% |
| ALFF | 173 | Left | Insular | 1.77% |
| ALFF | 211 | Left | Subcortical Nuclei | 1.74% |

**Note:** Node number is derived from parcellation in HBN atlas and percentage is calculated as the weight of a certain feature divided by the sum of weights for all 49 features.

**Abbreviation:** ALFF, amplitude of low frequency fluctuation; DC, degree centrality; GMV, gray matter volume; HBN atlas, human brainnetome atlas; NC, normal control; ReHo, regional homogeneity; SZ, schizophrenia.

**TABLE S9**

Feature ranking for FESZ vs CSZ classification with HBN atlas

| Feature | Node | Brain Side | Region | Percentage |
| --- | --- | --- | --- | --- |
| ReHo | 100 | Right | Temporal | 3.73% |
| DC | 61 | Left | Frontal | 2.82% |
| ReHo | 31 | Left | Frontal | 2.51% |
| ReHo | 92 | Right | Temporal | 2.50% |
| GMV | 54 | Right | Frontal | 2.44% |
| ReHo | 244 | Right | Subcortical Nuclei | 2.41% |
| ReHo | 45 | Left | Frontal | 2.37% |
| ALFF | 105 | Left | Temporal | 2.32% |
| GMV | 59 | Left | Frontal | 2.26% |
| ReHo | 46 | Right | Frontal | 2.18% |
| GMV | 32 | Right | Frontal | 2.17% |
| ReHo | 242 | Right | Subcortical Nuclei | 2.16% |
| GMV | 243 | Left | Subcortical Nuclei | 2.13% |
| GMV | 125 | Left | Parietal | 2.13% |
| ALFF | 237 | Left | Subcortical Nuclei | 2.12% |
| DC | 118 | Right | Temporal | 1.96% |
| GMV | 51 | Left | Frontal | 1.92% |
| GMV | 183 | Left | Limbic | 1.88% |
| GMV | 72 | Right | Temporal | 1.86% |
| DC | 104 | Right | Temporal | 1.82% |
| ReHo | 213 | Left | Subcortical Nuclei | 1.77% |
| DC | 243 | Left | Subcortical Nuclei | 1.74% |
| GMV | 139 | Left | Parietal | 1.74% |
| GMV | 31 | Left | Frontal | 1.71% |
| GMV | 163 | Left | Insular | 1.71% |
| ReHo | 124 | Right | Temporal | 1.71% |
| DC | 5 | Left | Frontal | 1.70% |
| ReHo | 94 | Right | Temporal | 1.66% |
| ReHo | 167 | Left | Insular | 1.65% |
| ReHo | 223 | Left | Subcortical Nuclei | 1.64% |
| GMV | 69 | Left | Temporal | 1.64% |
| ReHo | 110 | Right | Temporal | 1.57% |
| ReHo | 114 | Right | Temporal | 1.54% |
| ALFF | 48 | Right | Frontal | 1.54% |
| ReHo | 48 | Right | Frontal | 1.51% |
| ReHo | 212 | Right | Subcortical Nuclei | 1.50% |
| GMV | 65 | Left | Frontal | 1.48% |
| ReHo | 12 | Right | Frontal | 1.48% |
| ReHo | 47 | Left | Frontal | 1.47% |
| DC | 236 | Right | Subcortical Nuclei | 1.41% |
| DC | 89 | Left | Temporal | 1.39% |
| ReHo | 75 | Left | Temporal | 1.39% |
| GMV | 10 | Right | Frontal | 1.38% |
| GMV | 64 | Right | Frontal | 1.36% |
| GMV | 129 | Left | Parietal | 1.36% |
| ReHo | 28 | Right | Frontal | 1.33% |
| ReHo | 30 | Right | Frontal | 1.31% |
| ReHo | 181 | Left | Limbic | 1.31% |
| GMV | 1 | Left | Frontal | 1.26% |

**Note:** Node number is derived from parcellation in HBN atlas and percentage is calculated as the weight of a certain feature divided by the sum of weights for all 49 features.

**Abbreviation:** ALFF, amplitude of low frequency fluctuation; CSZ, chronic schizophrenia; DC, degree centrality; FESZ, first-episode schizophrenia; GMV, gray matter volume; HBN atlas, human brainnetome atlas; ReHo, regional homogeneity.
